# Miniaturized NIR Spectroscopy in Food Analysis and Quality Control: Promises, Challenges, and Perspectives

**DOI:** 10.3390/foods11101465

**Published:** 2022-05-18

**Authors:** Krzysztof B. Beć, Justyna Grabska, Christian W. Huck

**Affiliations:** Institute of Analytical Chemistry and Radiochemistry, University of Innsbruck, Innrain 80-82, 6020 Innsbruck, Austria; christian.w.huck@uibk.ac.at

**Keywords:** food quality, food fraud, quality control, near-infrared, NIR sensors, miniaturization, handheld, portable, vibrational spectroscopy

## Abstract

The ongoing miniaturization of spectrometers creates a perfect synergy with the common advantages of near-infrared (NIR) spectroscopy, which together provide particularly significant benefits in the field of food analysis. The combination of portability and direct onsite application with high throughput and a noninvasive way of analysis is a decisive advantage in the food industry, which features a diverse production and supply chain. A miniaturized NIR analytical framework is readily applicable to combat various food safety risks, where compromised quality may result from an accidental or intentional (i.e., food fraud) origin. In this review, the characteristics of miniaturized NIR sensors are discussed in comparison to benchtop laboratory spectrometers regarding their performance, applicability, and optimization of methodology. Miniaturized NIR spectrometers remarkably increase the flexibility of analysis; however, various factors affect the performance of these devices in different analytical scenarios. Currently, it is a focused research direction to perform systematic evaluation studies of the accuracy and reliability of various miniaturized spectrometers that are based on different technologies; e.g., Fourier transform (FT)-NIR, micro-optoelectro-mechanical system (MOEMS)-based Hadamard mask, or linear variable filter (LVF) coupled with an array detector, among others. Progressing technology has been accompanied by innovative data-analysis methods integrated into the package of a micro-NIR analytical framework to improve its accuracy, reliability, and applicability. Advanced calibration methods (e.g., artificial neural networks (ANN) and nonlinear regression) directly improve the performance of miniaturized instruments in challenging analyses, and balance the accuracy of these instruments toward laboratory spectrometers. The quantum-mechanical simulation of NIR spectra reveals the wavenumber regions where the best-correlated spectral information resides and unveils the interactions of the target analyte with the surrounding matrix, ultimately enhancing the information gathered from the NIR spectra. A data-fusion framework offers a combination of spectral information from sensors that operate in different wavelength regions and enables parallelization of spectral pretreatments. This set of methods enables the intelligent design of future NIR analyses using miniaturized instruments, which is critically important for samples with a complex matrix typical of food raw material and shelf products.

## 1. Introduction

During the last four decades, near-infrared (NIR) spectroscopy (800–2500 nm; 12,500–4000 cm^−1^) has become one of the most attractive and used methods for food analysis and quality control for the following reasons: it represents a nondestructive analytical tool that allows a fast and simultaneous qualitative and quantitative characterization of a wide variety of samples with regard to their chemical compositions and physical attributes [1,2,3]. NIR spectroscopy is nowadays seen as a critical element to be successfully integrated into the modern system for food monitoring on its path to sustainability [4]. The last decade marked a rapid acceleration in the continuing trend of the miniaturization of NIR spectrometers. These devices significantly increase the flexibility of analysis; however, attention needs to be paid to the various factors that affect their performance in different scenarios [1,2,3,4,5,6]. Currently, it is a focused and very active research direction to systematically perform evaluation studies of the analytical accuracy and reliability of various miniaturized spectrometers available in the market [5,7].

NIR spectroscopy is a particularly potent tool for analyzing whole foods and highly processed products and their constituents [8,9,10,11,12,13,14]. The challenging characteristics of such analyses are commonly encountered in the agri-food sector [12,15,16] and the medicinal plant sector of the pharmaceutical industry [17,18,19], as well as in environmental monitoring and ecology studies [20,21,22,23]. As the result of chemical diversity and often challenging physical properties as well, such as the specific surface texture, the presence of layers resulting from biological structure of plant tissue [24] (e.g., husks), and even the presence of color, miniaturized NIR instruments often face serious challenges in the analysis of chemically complex, granular, and inhomogeneous samples typical for food items. Therefore, the applicability and analytical performance of miniaturized NIR spectrometers in such applications is the topic of extensive feasibility research, a factor of critical importance for establishing practical applications with high reliability.

It is noteworthy that the development of analytical applications of NIR spectroscopy originated from the needs of the agri-food sector, and its subsequent evolution was largely stimulated by the needs of the analyses required therein [11]. The decisive advance of this technique toward wide adoption was tightly coupled with the progress in the instrumentation, where the appearance of Fourier-transform (FT)-NIR spectrometers in early 1990s was the first major cornerstone [25,26]. The second breakthrough in technology can be easily associated with the introduction of portable and handheld instruments in the 2000s. However, from the conceptual point of view, the step into portability formed a much more decisive reshaping of the application horizon of this technique, as that breakthrough was the cornerstone of transiting the analysis from the lab to the site, which brought particular benefits to the agri-food sector [5].

**Figure 1:**
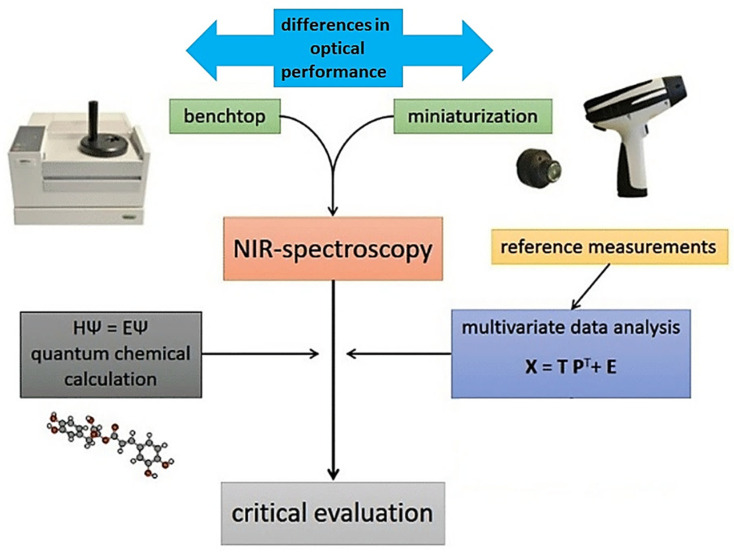
The workflow of the multiplanar methodology used for understanding the instrumental performance and increasing the accuracy, flexibility, and applicability of miniaturized NIR spectroscopy.

Applications of miniaturized NIR spectroscopy in food-related scenarios connect to a variety of problems, in which the design and knowledge-based optimization of an analytical pathway is essential to maximize the practical gain from using this innovative technology (Figure 1). In this review article, we present an overview of the current research directions, with a critical inspection of the key elements constituting the advancement of the micro-NIR analytical framework for modern food analysis, quality control, and safety risk monitoring. In particular, attention is given to the importance of using combined tools integrated into the NIR analytical method, which improve its accuracy, reliability, and applicability. Advanced methods of calibration (e.g., artificial neural networks (ANNs)) directly improve the performance of miniaturized instruments in analyzing complex and challenging samples, equalizing the accuracy of these instruments with benchtop spectrometers. Two-dimensional correlation spectroscopy (2D-COS) yields insights into the relative sensitivity observed between different instruments toward specific NIR bands [27]. NIR spectra are intrinsically very complex with highly convoluted signals, which becomes an extremely strong feature in the case of complex, multiconstituent samples, such as those of natural origin [28,29]. Quantum-mechanical simulations of NIR spectra provide deep comprehension of the chemical information in the processed spectra. This method unveils the chemical structures correlated with meaningful features of calibration models [30]. These simulations also enable the interpretation of the instrumental differences observed between different handheld sensors in light of the chemical information of a given constituent, and to the prediction of the performance level of a spectrometer in a similar analysis. With the comprehension of the chemical information analyzed by each spectrometer, the effectiveness of the sensor-fusion approach can be further validated. Finally, calibration transfer enables sharing of the trained calibration models between various NIR sensors, providing a large practical gain in efficiency. This suite of methods enables a better-informed design of future NIR spectroscopic analyses, which is particularly important for most samples with a complex matrix (Figure 1) [31].

## 2. Modern NIR Instrumentation—Toward Sensor Ultraminiaturization and Integration

Much of the specifics of the analytical framework based on miniaturized NIR spectrometers may be better comprehended by highlighting the decisive differences that exist between a standard laboratory stationary (i.e., benchtop) NIR spectrometer and handheld instruments. The former is nowadays a fully matured instrument that follows a rather uniform construction scheme, in which either a Michelson or a polarization interferometer is used, and the spectrum is acquired based on the Fourier-transform (FT) principle [32]. In contrast, there are numerous optical and engineering principles implemented in the competing portable and miniaturized NIR instruments, constituting the diversity in the operational characteristics, price-per-unit factor, and the ultimate applicability of these devices.

### 2.1. General Design of an FT-NIR Benchtop Spectrometer

A construction scheme of a benchtop NIR spectrometer does not essentially differ from that of a generic instrument for optical absorption spectroscopy [33,34,35]. It comprises a light source, a wavelength selector, and a detector as the main building/functional blocks, which are interconnected by optics for the propagation of the beam. While NIR spectrometers can be straightforwardly configured for transmission measurements, in which case a sample compartment is typically integrated within the spectrometer’s casing, a diffuse reflection mode of spectra acquisition is much more popular in analytical applications. This advantage results from the physical principles of NIR spectroscopy (i.e., high permeability of typical organic matter against NIR wavelengths), which make it straightforwardly applicable for obtaining good-quality reflectance spectra of samples without any prior pretreatments, such as dilution in a nonabsorbing medium (e.g., KBr powder), which is necessary for measuring mid-infrared (MIR) spectra, with the cost of a destructive method of analysis. Unlike most other instruments, NIR spectrometers can be readily equipped with fiber probes for remote scanning in diffuse reflectance mode, further adding to the versatility and practical usefulness of NIR spectroscopy.

An NIR spectrometer can be implemented using two different concepts of performing the wavelength selection. In a dispersive spectrometer, at a given time, only a narrow waveband is passed through a monochromator (e.g., a diffraction grating and optical slit system) and subsequently presented to the detector. The motion of the diffraction grating over time selects a consecutively changing waveband (i.e., narrow fragment of the wavelengths), effectively scanning the entire spectral region in which the instrument operates. As mentioned in the introduction, this mode of operation has been made obsolete in benchtop NIR spectroscopy by a superior FT spectrometer [33,34]. In an FT-NIR instrument, the entire measured wavelength region (i.e., broadband) is passed to the detector. Using an interferometer, either a Michelson-type or the less-popular polarization interferometer, an interferogram (i.e., a signal in the time domain) is registered by the detector. The signal in the frequency domain (i.e., the spectrum) is reconstructed from the interferogram through a Fourier transform. The primary benefits of such a solution are the gain in the optical throughput of the spectrometer and a precisely controlled wavelength axis [33]. In contrast to full-scale benchtop spectrometers, miniaturization introduces several difficulties that effectively reduce the advantage of implementing the FT principle in the spectrometer [5]. Certain other solutions, including multichannel devices, are feasible and offer competitive performance and cost-effectiveness. Consequently, the handheld instruments available on the market utilize diverse optical principles and engineering solutions to acquire NIR spectra [5].

### 2.2. Functional Design Scheme of a Miniaturized NIR Spectrometer

#### 2.2.1. Radiation Source

A tungsten halogen incandescent lamp is almost always used as a light source in benchtop NIR spectrometers. With a few exceptions, it is also a standard used in miniaturized devices [14]. The emission profile of this source makes it very well suited for the NIR region, creating a simple, reliable, bright source with very good stability when thermal equilibrium is achieved. The tungsten halogen source is also preferred in miniaturized NIR spectrometers; however, there are additional requirements to make it suitable for such implementation. Energy efficiency and physical dimensions must be optimized, and the thermal stability of the source can become a problem in miniaturized devices due to a limited heat capacity or potential exposure to environmental conditions (e.g., sunlight) during field operation. For example, the insufficient thermal stability of some of earlier designs of otherwise very potent miniaturized instruments was found to negatively affect their analytical performance; as reported in a case study, additional cooling of the entire instrument with a thermocouple eliminated this shortcoming [36]. The effect of source heating on the entire miniaturized spectrometer can be minimized by supplying the source with power only for the duration of the measurement; in some designs, this occurs automatically. Other solutions include a temperature-correction function implemented in spectrometer software, such as that for the MicroNIR 1700 ES instrument. Regardless, it is recommended that the background and dark scan are collected frequently to keep the background profile up to date during measurements.

On the other hand, a light-emitting diode (LED) is a semiconductor source that offers an extremely low power consumption and operating voltage, compactness, and durability, and has an excellent value compared to its cost [37]. However, current technology offers LEDs that emit in a relatively narrow wavelength range that only partially covers the NIR region; for example, a gallium arsenide (GaAs) LED has a bandwidth of only 50 nm with a maximum emission at 870 nm [38]. Consequently, these components are suitable for use in visible/short-wavelength NIR (Vis/SW-NIR) spectrometers, in which compact dimensions and cost-effectiveness of the instrument are crucial (e.g., SCiO) [5].

#### 2.2.2. Wavelength Selector

The wavelength-selection principle and its corresponding implementation in hardware are the most critical characteristics of a spectrometer, and largely determine its overall design and operating parameters [5]. Furthermore, this element manifests the widest diversity among the designs present in the market, making it the most essential for the characterization of a given instrument. Although interferometer-based designs dominate in benchtop spectrometers, implementation of a Michelson interferometer in handheld devices involves considerable trade-offs resulting from difficulties in miniaturizing this complex element [5]. Consequently, it is no longer far superior to other solutions, and so far no uniformly “best” concept for wavelength selection has been established for a miniaturized NIR spectrometer. The currently available portable NIR spectrometers demonstrate far-reaching diversity in this regard, with wavelength selectors ranging from the Fabry–Pérot interferometer and the Hadamard mask to multichannel devices that combine a linear variable filter (LVF) with an array detector. On the other hand, miniaturized dispersive spectrometers have been proved to be competitive; e.g., those implementing a digital micromirror system that avoids the use of movable dispersion grating, and thus are well suited to the regime of miniaturization [5]. Complex wavelength selectors allow the use of single-element detectors, resulting in the most cost-effective combination; the alternative is the integration of an expensive array detector with a fairly simple optical filter. Several known types of wavelength selectors are subject to miniaturization using microelectromechanical system (MEMS) or micro-optoelectro-mechanical system (MOEMS) technology [39]. These optomechanical devices are assembled with silicon using industry-standard technologies for the production of integrated circuits, and their popularization had a particularly important role in the development of miniaturized NIR spectrometers [5].

The principle of the Hadamard transform (HT) as the wavelength-selection approach was implemented in multiple handheld NIR spectrometers [40]. The practical advantages of Hadamard NIR spectrometers were discussed in detail by Fateley and co-workers [41,42]. In its simplest form of the single-encoded HT spectrometer, the light beam is focused on a slit, and after passing through the grating and the associated optics, is encoded by a multiaperture mask (Hadamard mask) and projected onto a single-pixel detector. This optical configuration results in a Hadamard-encoded signal reaching the detector and the spectrum being restored through a Hadamard transform. Theoretically, the advantages of Hadamard spectrometers were demonstrated relatively early, as they shared optical benefits with FT instruments; namely, the multiplex (Felgett), frequency accuracy (Connes), and throughput (Jacquinot) advantages, while HT spectrometers do not extensively rely on moving parts [40]. Importantly, a programmable Hadamard mask proved to be implementable via MOEMS technology, contributing largely to the success of this solution in handheld NIR instruments.

**Figure 2:**
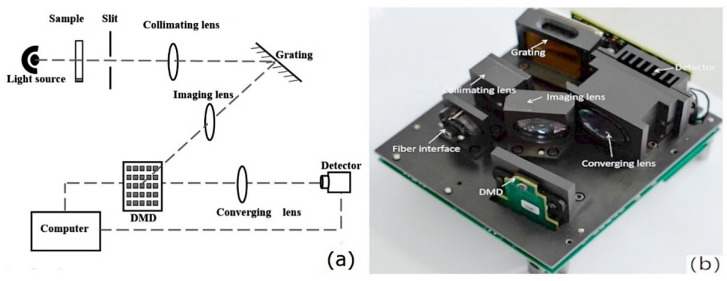
The implementation of a Hadamard-transform (HT) spectrometer by Lu et al. (**a**) Scheme of the optical system; (**b**) design of HT spectrometer. Reproduced (CC-BY 4.0 license) from [43].

A digital micromirror device (DMD) is a wavelength selector in which an array of microscale mirrors manufactured using MOEMS technology form the wavelength-scanning element. The implementation of a DMD enables the construction of a dispersive spectrometer in which this element is accompanied by a fixed dispersive grating instead of the moving grating characteristic of the canonical optical spectrometer [5]. DMD design also enables the construction of a Hadamard-transform spectrometer (Figure 2) [43]. In contrast to that obsolete design, a DMD-based spectrometer has no moving macroparts and offers advantages in terms of mechanical robustness, size, and cost-effectiveness of the wavelength selector element itself, while its optical configuration allows the use of an inexpensive single-pixel detector.

The Fabry–Pérot interferometer also acts as a miniaturized wavelength selector [5]. Its scheme of operation is based on two parallel mirrors separated by a constant or variable distance, forming a Fabry–Pérot cavity. The filter is transparent only for resonant wavelengths related to the standing wave effect of the electric field generated in the optical resonator and controlled by the cavity width. Variable filter settings allow incoming polychromatic band (i.e., broadband) to be successively divided into several narrower wavelength fragments (i.e., narrow bands). Microfabrication of a programmable Fabry–Pérot interferometer is also feasible using MEMS technology. It is possible to easily reconfigure the spectrometer to work in other spectral ranges as a factory setting; for example, in NIRONE sensors.

Furthermore, while not being clearly superior in miniaturized form, the Michelson interferometer has been implemented in a number of compact NIR instruments, including those that achieved considerable commercial success; e.g., NeoSpectra sensors [5]. Notably, a more recent generation of miniaturized FT-NIR spectrometers has emerged; for example, the Hefei SouthNest spectrometer. This design implements a relatively large mirror with a diameter of 3 cm in the interferometer, resulting in much improved optical throughput of the spectrometer compared with earlier generations of miniaturized FT-NIR devices. Michelson-interferometer-based sensors offer a wide operational spectral region and superior resolution, comparable to those offered by benchtop FT-NIR spectrometers.

In addition to the above examples, one alternative concept for a spectrometer constitutes an array detector with multiple independent photosensitive elements [5]. While expensive, such a detector only needs to be combined with a relatively simple optical filter to work efficiently as a multichannel spectrometer that measures all wavelengths in the spectrum simultaneously without any scanning principle involved. This solution is particularly beneficial for miniaturized instruments, as it involves no moving parts even at the microscale, a high mechanical resistance, and very compact dimensions. Among the solutions implemented in the current miniaturized NIR instruments, multichannel sensors based on a linear variable filter (LVF) deserve particular attention. An LVF works as a wedge-shaped optical filter with an optical coating of a different thickness, which creates a linear variation in the transparency of the filter at different wavelengths. Designs based on an array detector and an LVF, unlike those based on MOEMS, do not have the high initial investment costs that are characteristic of semiconductor manufacturing. An LVF element is very thin itself, allowing the construction of instruments with a very short path length, further improving the properties of the multichannel spectrometer with high optical performance. Since there is no movement in the operation of the spectrometer, acquisition of a single spectrum is possible with an integration time of less than 10 ns, resulting in the ability to average a large number of spectra in an overall short collection time [5].

#### 2.2.3. Detector

**Figure 3:**
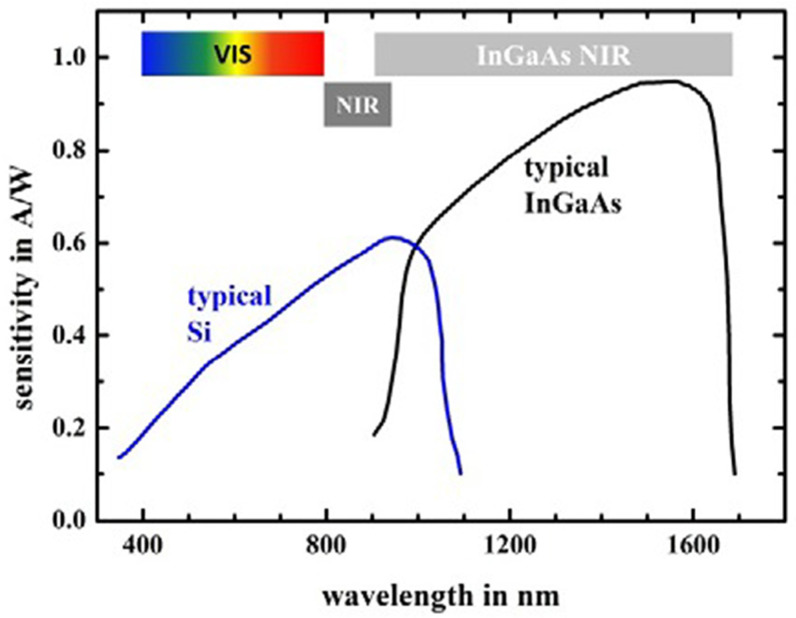
Typical quantum efficiency spectra of Si and InGaAs detectors. The colored and grey bars indicate the visible, SW-NIR, and conventional NIR (named “InGaAs NIR” in the source) wavelength ranges. Reproduced (CC-BY 4.0 license) from [45].

Miniaturized NIR spectrometers are usually equipped with an indium gallium arsenide (InGaAs) or “extended” InGaAs detector, although some instruments also contain silicon (Si) photovoltaic diodes [5,34,44]. The size constraint seen in miniaturized spectrometers limits their optical performance. Therefore, in order to maintain an adequate signal-to-noise (S/N) ratio, the InGaAs detector is more desirable due to its high sensitivity, especially in the range of the longer wavelengths of the NIR region (Figure 3) [14]. The typical wavelength range for its optimal performance is around 1000–1600 nm (10,000–6250 cm^−1^); however, in practice, several InGaAs-based NIR spectrometers offer good performance at adjacent wavelengths as well. Compared to other types of detectors, InGaAs detectors offer a fast response time, good quantum efficiency, and low dark current, allowing a short scan time while maintaining a good S/N [5]. The extended InGaAs detector is suitable for instruments operating at shorter wavelengths of ca. 1700 nm. However, this type of detector has a lower sensitivity, and may require integrated cooling solutions [44].

Photovoltaic Si detectors maintain a reasonable sensitivity in the wavelength range from the visible region to ca. 1100 nm (9100 cm^−1^), which makes them suitable for cost-effective, compact spectrometers operating only in the visible and SW-NIR regions, as presented in Figure 3 [5,44]. Photodiodes used in portable spectrometers require the use of a wavelength-blocking filter to mask the detector from sunlight. The favorable affordability of this type of detector makes it particularly suitable for spectrometers oriented toward the consumer market [5,44].

#### 2.2.4. Other Elements

**Optics**. NIR spectrometers are compatible with glass optics because this material does not absorb in the visible and most of the NIR region [5,34]. This enables the use of cheap mechanically and chemically resistant optical materials for the construction of portable NIR spectrometers [5,44]. However, the best performance in the long-wavelength part of the NIR region may require high-quality optics made from fused silica; i.e., without O-H impurities. To ensure reliable operation in direct contact with the sample, the optical window at the sample interface made from a scratch-resistant material is preferred. For example, some designs employ sapphire for this role, as it is a mechanically resilient material with the required transparency in the NIR wavelength range. However, it features a rather high refractive index (greater than 1.7 in the visible and NIR regions) that increases optical loss from reflection, making it more suitable for instruments with good optical throughput, such as MicroNIR multichannel spectrometers.

**Connectivity, user interface, and power delivery**. Modern electronics have achieved high levels of energy efficiency, which is a great advantage for portable spectrometers. These instruments follow one of two power delivery concepts: either the power supply is provided by an external source, or the unit is equipped with its own battery. For many spectrometers, the first solution is practically implemented with a universal serial bus (USB) connection, which is also used for spectrometer control and data transfer (for example, the standard version of the MicroNIR spectrometer). However, this is only possible if the total power consumption of the instrument does not exceed the capacity of the USB interface. In addition, the use of the instrument is limited by a permanent connection to the main computer (PC) via a USB cable. The second solution is needed for completely autonomous spectrometers (e.g., microPHAZIR) and those compatible with smartphone applications (e.g., Tellspec Enterprise sensor and SCiO). With this latest type of device, the data interface for transferring measured spectra and associated data can be maintained via USB as well (e.g., microPHAZIR) or via a cloud service (e.g., Tellspec Enterprise sensor and SCiO). 

Closely related to the above, the user control over the instrument can also be achieved in several ways. The fully autonomous instruments feature their own user interface with a display screen and a user-input device (e.g., a keyboard), as in the case of, for example, microPHAZIR. Spectrometers that require continuous external power are typically controlled by a PC-installed application with the data interface and power delivery conveniently handled by a wired USB interface; examples include MicroNIR instruments [5]. Many devices aimed at the consumer market are operated through an application installed on a smartphone with continuous communication with a user device over a wireless connection; i.e., Wi-Fi or a low-power Bluetooth interface. 

### 2.3. Brief Overview of Selected Representative Miniaturized NIR Spectrometers

The principle of the Hadamard spectrometer was implemented in one of the first handheld NIR instruments introduced to the wide market by Polychromix, now the intellectual property of Thermo Fisher Scientific Inc. The instrument employed a programmable microscale MEMS-based Hadamard mask, a low-power tungsten lamp source, and an InGaAs single-element detector. These solutions enabled a robust, reliable, and reasonably compact instrument, given its fully autonomous operation. The device was fitted with its own power source—a lithium-ion battery—that was swappable for continued operation, a display screen, and a user interface; i.e., a keyboard. 

Several successful products on the market are based on the NIRscan Digital Light Processor (DLP) module from Texas Instruments. This solution is based on a digital micromirror device (DMD) manufactured using MEMS technology, and is available as two evaluation modules (EVMs): a high-performance (HP) EVM with a DLP NIRscan sensor and a mobile sensing (MS) EVM with a DLP NIRscan Nano. The latter, more compact one is primarily suitable for cost-efficient portable spectrometers. It is implemented in the NIR-S-G1 instrument from InnoSpectra [46], available as a customized product from, e.g., SphereOptics [47], Sagitto [48], Allied Scientific [49] and Tellspec [50]. The NIR-S-G1 spectrometer is extremely compact (82 mm × 63 mm × 43 mm; weight less than 145 g); it is equipped with li-ion battery, is operated through a mobile app, and communicates with a smartphone via a power-efficient Bluetooth interface.

A Fabry–Pérot interferometer was implemented by Spectral Engines in a miniaturized NIR spectrometer NIRONE S sensor [51], with several variants preconfigured for different operational wavelength-range, S/N ratio, and resolution parameters (Table 1) while maintaining very compact dimensions (25 × 25 × 17.5 mm; weight 15 g). The implementation of the Fabry–Pérot interferometer created an optical configuration of the sensor suitable for detection in a relatively large area of either the InGaAs or extended InGaAs type. The Sensor X is a compact version of the instrument optimized for cost-effectiveness and ease of production. Notably, the latest advances in Fabry–Pérot interferometer technology show promise for ultraminiaturization. For instance, Hamamatsu recently unveiled a series of ultracompact NIR sensors, differing mostly in their operational spectral regions that, however, are quite narrow and depend on the variant, ranging from 1350 to 2150 nm (7407–4651 cm^−1^) [52]. 

An alternative approach to a multichannel spectrometer is offered by the VIAVI MicroNIR series of instruments. These devices combine a multielement array detector (InGaAs) coupled with an LVF, enabling a very compact, mechanically robust spectrometer with superior optical performance for its size. Newer versions of the MicroNIR; e.g., the 1700 ES, improve the operational stability over time thanks to a temperature-correction function, effectively recalibrating the detector’s response depending on its temperature to mitigate the thermal capacity imposed by the compact dimensions of the device. The standard MicroNIR instrument is powered and controlled via a wired USB connection with a host PC, while the dedicated OnSite-W variant, intended for in-field operation, is equipped with battery power source and a waterproof and dustproof housing [53]. 

**Figure 4:**
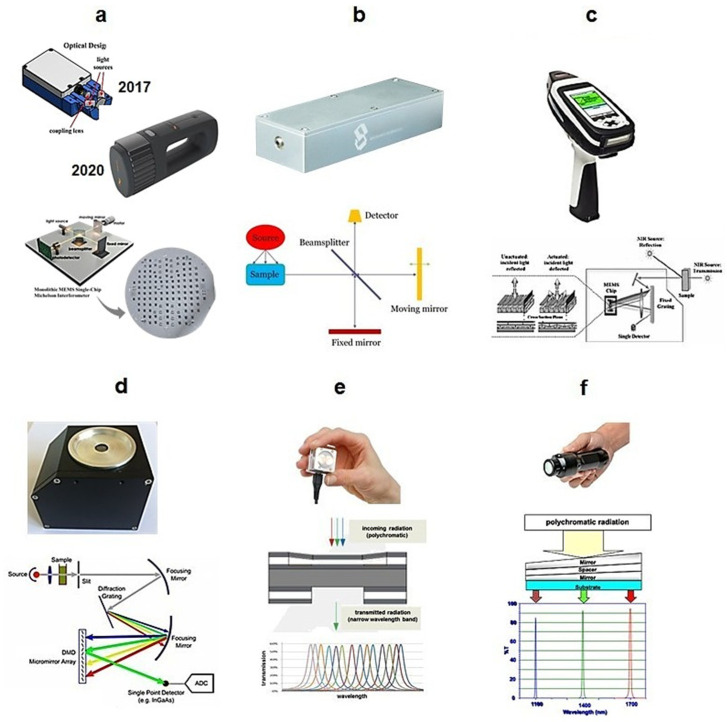
Principles of wavelength selectors built into different handheld NIR spectrometers: (**a**) MEMS Michelson interferometer—NeoSpectra, Si-Ware, Cairo, Egypt; (**b**) MEMS Michelson interferometer with a large mirror—nanoFTIR NIR, SouthNest Technology, Hefei, China; (**c**) MEMS Hadamard mask—microPHAZIR, Thermo Fisher Scientific, Waltham, MA, USA; (**d**) MEMS DMD—implementation of DLP NIRscan module, Texas Instruments, Dallas, TX, USA; (**e**) MEMS Fabry–Pérot interferometer—NIRONE Sensor S, Spectral Engines, Helsinki, Finland; (**f**) LVF—MicroNIR Pro ES 1700, VIAVI, Santa Rosa, CA, USA. Adopted (CC-BY 4.0 license) from [7].

Miniaturized FT-NIR spectrometers equipped with a Michelson interferometer are offered by, e.g., Si-Ware Systems with a NeoSpectra device (Table 1, Figure 4) [54]. Recently, a new generation of FT-NIR minispectrometers appeared. For example, Hefei SouthNest Technology introduced the nanoFTIR NIR spectrometer, which operates in the full NIR range of 800–2600 nm (12,500–3846 cm^−1^) while maintaining a relatively high spectral resolution of 6 nm at 1600 nm. The device has compact dimensions (14.3 × 4.9 × 2.8 cm) and is light (220 g), and can be equipped with an external light source and a fiber-optic probe compliant with the industry standard, making it suitable for online analysis. Furthermore, recently another MEMS-based FT-NIR spectrometer from Hamamatsu appeared, equipped with a large mirror (3 mm diameter) interferometer, enabling a good S/N ratio with a wide spectral range of 1100–250 nm (9090–4000 cm^−1^). On the other hand, regardless of the underlying technology, many of the spectrometers are offered in a specialized variant sold as “turn-key” analyzers to be operated by personnel not trained in spectroscopy. Such analyzers are preconfigured for the intended analyses, with a specialized software suite containing spectra-processing algorithms and precalibrated models for quantitative and qualitative analyses typically performed in a given area of application. For example, several analyzers based on the microPHAZIR spectrometer appeared that are intended to be operated under minimal supervision [55]. Exemplary configurations include, e.g., the microPHAZIR AG Handheld Analyzer, which is intended for animal feed analysis, and is preconfigured to predict major quality parameters and ingredients in these products, such as moisture, protein, fiber, starch, etc., [56]. The other turn-key configurations of the microPHAZIR include, e.g., the microPHAZIR PC analyzer, which is intended for plastics analysis in recycling [57]; the microPHAZIR RX analyzer, which is preconfigured for pharmaceuticals [58]; or the microPHAZIR AS, which fulfills the role of an asbestos analyzer [59]. Mini-NIR analyzers in particular are growing in popularity in the agri-food industry, with several examples of specialized devices; e.g., the NIR4 Farm spectrometer from AB Vista, which is intended for the analysis of feed and forage [60]; as well as analyzers that are preconfigured for grain assessment, such as AURA’s Handheld NIR [61] or the X-NIR Analyzer [62].

In the context of food analysis, attention should be given to specialized, consumer-oriented NIR spectrometers designed to accept somewhat limited overall performance with the greatest benefit of cost-effectiveness. These are the necessary trade-offs to fit the instruments into their specific niche of the market, where they are offered as “pocket food analyzers” for use by the general public [63]. The sensor hardware is tailored to provide a sufficient optical performance, while the primary value for the intended operators originates from the associated software. These instruments offer an easy-to-use cloud service, in which the results of the analysis are displayed to the end user in a “black-box”, with underlying predictions based on precalibrated models stored in the cloud service. A good example of such a device is the Consumer Physics SCiO NIR microspectrometer [64]. Marketed as the first “pocket” spectrometer, the unit measures 67.7 × 40.2 × 8.8 mm, weighs 35 g, and is intended primarily for everyday consumer assessment of food quality and nutritional value. The necessary economical affordability is achieved by using an LED light source and a simple 12-element Si photodiode detector, with an array of a 4 × 3 configuration, combined with optical filters across each pixel to form a 12-channel spectrometer. However, in this design, noticeable penalties in terms of optical performance were inevitable, manifested primarily in the low number of measured wavelengths. Below-average S/N levels and a narrow wavelength range covering only a fragment of the visible/SW-NIR range (740–1070 nm; 13,514–9346 cm^−1^) seem sufficient for the intended applications of this device, as a number of essential quality parameters of foods can be effectively predicted from these spectra. In connection with the ultraminiaturized, consumer-oriented instruments described above, the much-anticipated NIR sensor fully integrated with a smartphone has remained a vivid concept in the past decade [65]. While the initial prototype revealed highly promising characteristics [66,67], so far, no such solution has appeared on the market. However, more recent advances into ultraminiaturization; e.g., as demonstrated by Hamamatsu MEMS-FPI spectrum sensors [65], suggest that NIR spectrometers integrated with smartphones may become commercially available in the next few years.

## 3. Methods and Techniques for Spectral Acquisition, Data Analysis, and Interpretation

### 3.1. Techniques for Spectra Acquisition

**Figure 5:**
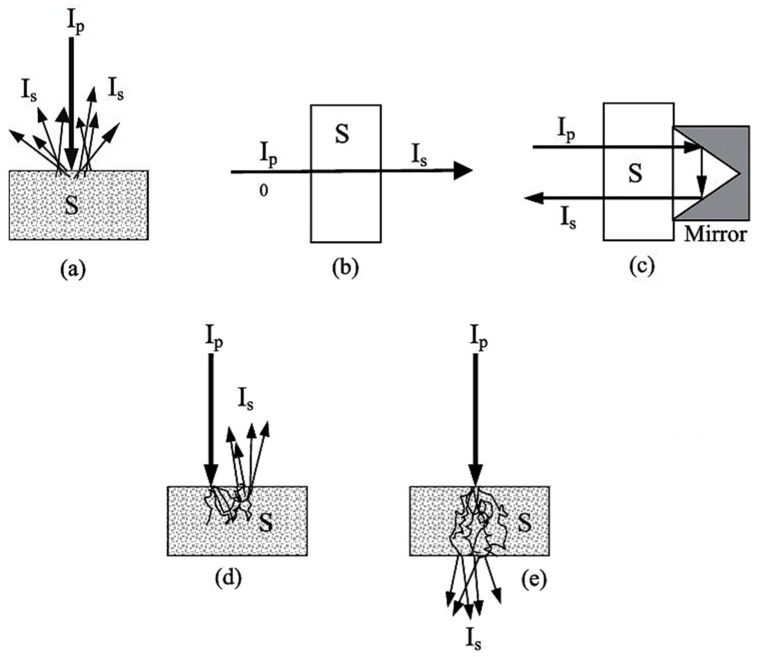
Modes of spectral acquisition employed in NIR spectroscopy: (**a**) diffuse reflectance; (**b**) transmittance; (**c**) transflectance; (**d**) interactance; (**e**) transmittance through scattering medium. Adopted (CC-BY 4.0 license) from [68].

Miniaturized spectrometers, in general, can be adopted to perform well in all modes of spectral acquisition established in NIR spectroscopy; i.e., diffuse reflectance (Figure 5a) and transmittance (Figure 5b), as well as the mode effectively combining both of these; i.e., transflectance (Figure 5c) [68]. Furthermore, the interactance mode (Figure 5d) can be distinguished, which is based on a geometrical arrangement of the sensor vs. the sample surface, rather than a distinct optical phenomenon. This configuration reduces optical losses at the path to the sample surface; i.e., a higher portion of the incident beam effectively reaches the sample. Additionally, in the case of the transmittance measurement of materials in which strong scattering occurs (e.g., dense solid samples), a distorted behavior of the NIR beam when propagating through the sample may occur (Figure 5e).

That being said, most miniaturized NIR spectrometers are factory-configured to operate in diffuse reflectance mode, and some of them are offered with additional accessories for transflectance measurements, as in the case of, e.g., the VIAVI MicroNIR. Typically, spectral acquisition in this mode is performed by using an external reflector and holder designed for maximizing the optical gain of a specific instrument. Such an accessory may be also custom-made; e.g., a gold-plated surface with a geometrical shape tailored to a specific analysis [36]. On the other hand, to perform conventional transmittance measurements, which are preferable for reliability in the analysis of suitable samples such as liquids, miniaturized instruments require an accessory with its own radiation source. A transmittance accessory is therefore less common in miniaturized NIR spectroscopy, but is offered again by, for example, VIAVI, to fit their MicroNIR spectrometer. The interactance principle is typically implemented through a fiber probe accessory. Different architectures of optical probes are possible: either the illumination and collection path use the same light fiber, resulting in a single-fiber probe (SFP) configuration, or two independent fiber probes are dedicated to each beam, resulting in a multiple-fiber probe (MFP) configuration. While intensively used in industrial environments, only certain general-oriented miniaturized NIR spectrometers are solely designed to operate via a fiber probe; e.g., the Hefei SouthNest FT-NIR instrument. 

MVA groups various mathematical methods capable of correlating many variables at one time [69]. Every spectral point carries information about the sample, and effectively, the correlation function binds the property of a multicomponent sample with its vibrational spectrum in a many-parameter function. The machine-learning methods in the variants applied in chemistry are commonly known as chemometrics, and these are the main tools for quantitative and discriminant (i.e., qualitative) analysis in applied spectroscopy [69,70]. These methods may be roughly divided into the following categories.

Exploratory data analysis (EDA) groups techniques for data mining (i.e., cluster analysis and principal component analysis (PCA)) used to gain a general overview of the variance in the dataset and explore the statistical properties (i.e., distribution in multi-variate space) of high volume, complex data; e.g., sets of spectra.

Pattern-recognition (classification) techniques are used for separation (grouping) of the samples according to the statistical specificity of the sample set. Supervised classification methods include, e.g., linear discriminant analysis (LDA) or support vector machine (SVM) classification [71]. Clustering methods [72]; e.g., hierarchical cluster analysis or k-means clustering, are archetypical unsupervised machine-learning algorithms that are frequently used in the determination of the similarities between samples by grouping unlabelled datasets. Artificial neural networks (ANNs) are very potent supervised methods for performing classifications, particularly in challenging cases; e.g., those affected by instrumental noise and other perturbations. 

Regression analysis groups the methods used for quantitative prediction of a sample’s properties; e.g., quantification of chemical content (either one or a group of constituents) present in the sample. The most popular techniques include multiple linear regression (MLR), principal component regression (PCR), and partial least-squares regression (PLSR). Nonlinear regression methods (e.g., Gaussian process regression (GPR)) and ANNs can perform very well and improve the analytical performance of miniaturized sensors, in which a lower resolution and spectral region limit the amount of information available for the analysis [31,73].

The statistical parameters of trained classification or regression models need to be evaluated to assess their validity (i.e., ability to describe the variance not only in the calibration set, but also in general population) and predictive performance. The coefficient of determination (*R*^2^), root-mean-square of calibration (RMSEC), and root-mean-square error of cross-validation (RMSECV) describe the robustness of the model. The ultimate prediction power of the model is best assessed by evaluating the root-mean-square error of prediction (RMSEP) on the basis of an independent one, or preferably more test sets of samples. Finally, the level of detection (LOD) and level of quantification (LOQ) are essential parameters that indicate a sensor’s performance (in a specific analysis) toward low-concentrated constituents, which often receive most focus in certain applications. Furthermore, statistical parameters can be used to directly compared different approaches or various instruments being used; e.g., to describe the performance of the sensing device in different conditions (for example, configuration of the sensing interface).

Advancements in chemometrics, or even more broadly understood data science, are naturally beneficial to the applications of miniaturized NIR spectrometers, although this is a generally oriented trend that brings merit to analytical spectroscopy, rather than focused research oriented strictly at providing gains only for miniaturized sensors. Certain aspects of this progress, however, might be linked with proportionally greater benefits offered by this specific technology. Data-fusion concepts are useful to combine spectral information from several sensors operating in narrow wavelength regions, as well as to provide the possibility of performing parallelized spectral pretreatments (Section 5.2). Development and validation of precalibrated models stored in cloud services should be mentioned, where the robustness (i.e., universality) of the models is a critical factor; however, this development remains mostly the proprietary intellectual property of instrument vendors. On the other hand, recent attention in NIR spectroscopy has been increasingly directed toward the application of deep neural networks (i.e., deep learning or “deep chemometrics”) for prediction and classification purposes [74,75,76]. However, to date, only scarce literature has appeared that studied the potential benefits of applying deep-learning methods to reinforce the analytical framework of miniaturized NIR sensors [75]. However, it seems plausible that deep networks could provide benefits in processing challenging data sets; e.g., those of complex samples measured by ultraminiaturized NIR spectrometers. On the other hand, recent critical evaluations of the current state of data science related to the applications of miniaturized NIR spectrometers indicate urgent problems yet to be comprehensively studied [77]. Giusanni et al. pointed out the attention to the issue of the possible deterioration of miniaturized sensors over time, and the connected problem of the validity of the respective calibration models. Other concerns expressed in that study included the transferability of the models to future generations of sensors, which seems entirely legitimate as we approach the era in which the effort invested in the training and validation of models may outweigh the unit cost of inexpensive sensors. Furthermore, issues related to the automatization of the data transfer from the instrument to the user device were pointed out as well [77]. Therefore, there appear to be specific challenges and problems to be solved by data scientists and chemometricians that are directly related to the widespread use of miniaturized NIR spectrometers by nonexpert personnel.

### 3.2. Methods for Interpretation of NIR Spectra

While conventional methods of spectral interpretation provide generalized tables of assignments of the NIR bands representative of chemical constituents typically present in foodstuffs (Table 2; limited to the major classes of chemical compounds only), the identification of specific markers in NIR spectra is much less straightforward than it is, for example, in MIR or Raman spectroscopy [78]. This is the consequence of the intrinsic complexity of NIR spectra and the resulting difficulty in their direct interpretation, which remains a limiting factor regardless of the application. In contrast to MIR and Raman techniques, NIR spectroscopy has been hindered in forming a practically accessible synergy with computational chemistry [79]. In recent years, however, advances in the tools used in computational chemistry have created an opportunity to take a step beyond this barrier [28,80,81,82]. 

This led to considerable advances in the applicability of the methods of theoretical chemistry to NIR spectroscopy [83]. The accurate simulation of NIR spectra of reasonably large molecules largely improves our comprehension of NIR spectra, and offers an opportunity to take a step beyond this barrier. Quantum-mechanical simulations of NIR spectra of a variety of compounds are significant from the point of view of physiochemical and analytical spectroscopy. The examples range from basic molecules (alcohols, nitriles, carboxylic acids) [84,85,86,87] to complex molecules with importance in biophysical science (fatty acids, nucleobases) [88,89], materials science and industry [90], and analytical chemistry (vitamins, natural drugs, polyphenols, alkaloids, food adulterants) [91,92,93,94,95]. The simulated NIR spectra largely increase the level of detail in the band assignments compared to the one available in conventional methods of spectral analysis (Table 2).

**Figure 6:**
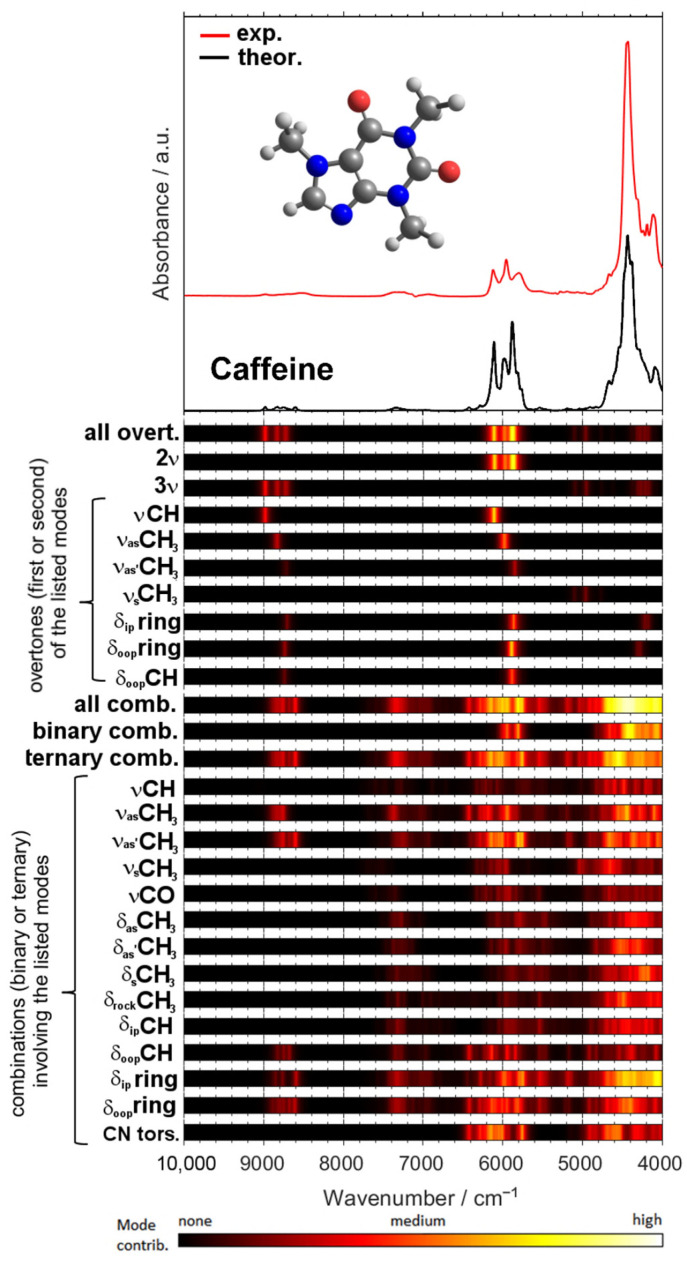
Vibrational modes’ contribution to NIR spectrum of caffeine available from quantum chemical calculations. Reproduced (CC-BY 4.0 license) from [93].

A remarkable potential arises from the growing applicability of anharmonic computations in solving the problems that arise in both basic and analytical NIR spectroscopy [96]. The highly convoluted, overlapping nature of NIR spectra can be successfully dissected in theoretical spectra, as presented in the example of a caffeine molecule (Figure 6) [93]. The elucidated rich information stemming from numerous NIR bands can subsequently be used to improve the basic understanding of NIR spectroscopy, as well as to advance its applications. In silico simulations of NIR spectra yield highly detailed and accurate chemical interpretations of the NIR bands. This information opens up new possibilities of performing a deep examination of the performance profile of handheld NIR spectrometers (Section 2.2 and Section 2.3). The calibration models constructed for different spectrometers capture chemical information on the analyzed constituent in clearly distinct ways, with the benchtop high-resolution spectrometer being able to capture individual vibrational bands much more accurately. This results in consequences to the ability of a spectrometer to acquire fine intensity changes in a specific task. The detailed comprehension of NIR bands from an accurate simulation of the spectra enables the knowledge-based design and optimization of analytical applications of NIR spectroscopy.

**Figure 7:**
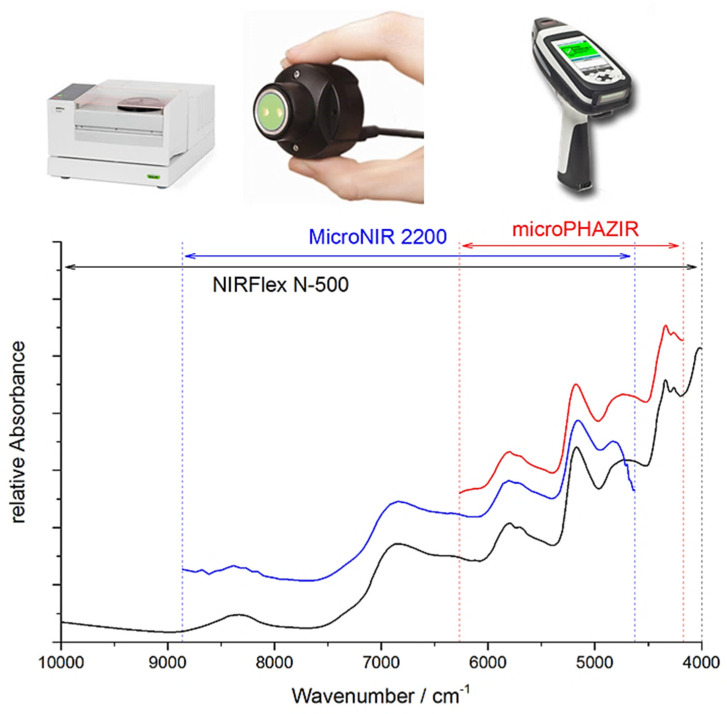
In contrast to benchtop FT-NIR instruments that measure the full NIR wavenumber region, the access of miniaturized NIR spectrometers to chemical information is selective, resulting from only narrow, and at best partially overlapping, fragments of the NIR spectra measured by these sensors. Adopted with the permission of the Royal Society of Chemistry (2017) from [27].

This detailed information on the chemical origin of each NIR band enables new approaches to the support of analytical applications. For example, one may select the best-suited sensor for the intended analysis by assessing its suitability in measuring the characteristic absorption regions of the targeted constituent. Miniaturized NIR spectrometers most often can only measure fragments of the NIR spectrum (Figure 7), and hence, only selective chemical information can be acquired by these devices. An accurate spectral simulation, such as the example for caffeine presented above (Figure 6), enable a full understanding of which NIR vibrations a specific sensor can acquire, and thus, the best-suited instrument for the targeted analysis can be selected. 

Furthermore, a detailed interpretation of PLS regression factors becomes possible as well, providing deep insights into the critically important connections between the chemical information present in the sample and the analytical framework [97]. In this way, the interpretation, in a chemical sense, of the meaningful variables in chemometric models becomes possible [98,99,100].

These three essential pillars, each of which takes advantage of a detailed understanding of the chemical information in the processed spectra, enable a “‘smart”, knowledge-based design and the optimization of an analytical approach in modern miniaturized NIR spectroscopy.

## 4. Overview of Applications of Miniaturized NIR Spectrometers in the Agri-Food Sector

NIR spectroscopy is one of the most versatile methods with great utility, and is highly valued as an analytical and quality-control tool for foods [101,102]. Miniaturized NIR spectrometers have particularly rich applications in agricultural and foodstuff areas, as their portability greatly enhances the common conventional strengths of the NIR analytical framework, and the miniaturized instruments meet the challenges and specifics of food-related analysis very well [11]. The complex nature of the food production and delivery chains, as well as the susceptibility of foods to quality loss, promote the need for a flexible analytical tool [103]. Similarly, onsite NIR spectroscopy is an excellent tool for monitoring the quality and growth conditions of crops, and the advent of this technology revolutionized certain aspects of agriculture [104]. For these reasons, mobile NIR spectrometers attracted relatively early attention in the area of food analysis [104,105]. Ellis and colleagues previously provided a perspective view on the specific capabilities of portable NIR devices and their applications in food supply chains [106]. 

The current state of the art of miniaturized NIR spectroscopy in food analysis shows that handheld instruments can be used successfully for a wide variety of problems, but the applicability potential and relative performance may vary from instrument to instrument [73,77,99,100,107,108,109,110,111,112,113,114,115,116,117,118,119,120,121,122,123,124,125,126,127,128,129,130,131,132,133,134,135,136,137,138,139,140,141,142,143,144,145,146,147,148,149,150,151,152,153,154,155,156,157,158,159,160,161,162,163,164,165,166,167,168,169,170,171,172,173,174,175,176,177,178,179,180,181,182,183,184,185,186,187,188,189,190,191,192,193,194,195]. In such applications, the instrumental difference can be profoundly manifested. For example, affordable visible/SW-NIR spectrometers can perform well in the analysis of macronutrients, but their applications in other scenarios may be limited [112,118]. This makes it difficult to predict the performance of a given spectrometer without performing systematic feasibility studies. Such attempts have been made, and it should be briefly mentioned that recent studies were aimed at obtaining a wider perspective on the instrumental differences manifested in the ability to acquire the characteristic signal of a specific constituent present in the sample [100]. Most of the recent research has focused on developing effective methods for determining the quality of shelf products. The challenges faced in this scenario result from several factors. Firstly, the chemical variety of foodstuffs, often further complicated by the complex matrix and high moisture content, are of note. Hence, for example, the accessibility to the spectral footprint of food adulterants in a specific spectral region may vary [94]. Next, the physical properties of these products, such as surface texture, may frequently interfere with the analysis. Finally, it is often desirable to perform a nondestructive analysis of an originally packaged product; i.e., the influence of the packaging material needs to be addressed. Consequently, the feasibility of miniaturized NIR spectrometers may greatly vary from case to case. Nevertheless, portable NIR spectroscopy has been introduced with remarkable success in the food industry. Application development remains an active direction of research in this area, and numerous reports appeared in the current literature, as summarized in the following sections.

### 4.1. Milk

Miniaturized NIR spectroscopy finds particularly widespread use in the analysis of dairy products, with a considerable research effort oriented directly at developing analytical methods for the analysis of milk, as evidenced by the recent literature [107,108,109,110,111,112,113,114,115]. Table 3 presents summarized and tabularized key information provided in the reviewed studies. Investigations toward the qualitative assessment of milk have attracted the main attention, where examples of discrimination between organic and conventional milk, authenticity checks and detection of milk adulteration, or discrimination between regular and lactose-free milk directly in the field should be noted. However, studies demonstrating the full capacity of miniaturized NIR spectroscopy to perform rapid quantitative predictions of the key quality parameters of milk, such as fat and protein content or fatty-acid composition, have been conducted as well.

Very often, the prediction performance offered by miniaturized NIR sensors in the established analytical framework was deemed comparable to that of benchtop NIR spectrometers, enabling the real-time monitoring of quality-control parameters of cow milk for each specimen. Furthermore, while primary attention is given here to bovine milk, examples of goat milk analysis can also be found in the recent literature as well [108]. In the area of methodology, it should be pointed out that there has been development toward an effective calibration transfer in order to share calibration models among several portable instruments, which provided decisive gains in efficient in situ analysis at the farm level [110].

### 4.2. Other Dairy Products

Dairy products other than milk also have been extensively examined by miniaturized NIR spectrometers, with numerous feasibility studies and practical applications of the developed methods. In this area, the analysis of cheese quality predominates, while examples of successful examinations of yogurts and milk/dairy powders can be provided in this category as well [116,117], as summarized in Table 4. Notably, good analytical figures of merit were accomplished in these feasibility studies; even in the cases of extremely cost-effective NIR sensors intended for the consumer market; the prediction performance remained acceptable even when cloud-based “black-box” calibration integrated with the consumer-aimed software was used for the analysis. Furthermore, the literature suggests that improved control of the cheese-making process is permissible with miniaturized NIR sensors, as these devices enable the early detection of deviations from the target quality directly in the production process, as well as the aging of cheese. On the other hand, the relevance of the visible/SW-NIR region to provide information correlated with the quality parameters of dairy products emerged from the reviewed studies. This seems to explain why these ultra-cost-effective sensors, which operate in the visible/SW-NIR region as the result of their constructional principles (i.e., Si photodetectors and LED sources), generally present very good performance in these applications when compared to benchtop NIR spectrometers that operate in the conventional NIR spectral range.

### 4.3. Meat

An inspection of the current literature found intensive development of meat analysis using miniaturized NIR spectroscopy (Table 5). Predominantly, attention is drawn toward the rapid, nondestructive analysis of freshness, which is a major concern in the everyday consumption of meat, as well as the authenticity check and the detection of adulteration, following several episodes of the mislabeling of meat that occurred in the past [9,11,13,128]. 

Notably, it was demonstrated that, in the case of the former concern, the aging day and levels of chemical/microbial indicators (i.e., thiobarbituric acid, volatile basic nitrogen, and bacteria levels) could be successfully analyzed using miniaturized NIR instruments in a rapid manner with no need for destructive sampling procedures [135]. On the other hand, classification of chicken meat by a portable NIR spectrometer while also discriminating between different parts of the chicken could be performed directly in the processing line.

Quantitative predictions often focus on the analysis of the fat content in meat, as well as the microbiological status, or the quantitative prediction of the level of fat adulteration [140]. Furthermore, quantification of a specific type of meat in a ground meat blend; e.g., the beef content in chicken/beef, pork/beef, and chicken/beef/pork blends, was successfully accomplished with miniaturized NIR spectroscopy as well [138]. In a number of investigations, the applicability of miniaturized NIR spectrometers in tackling the challenging problem of the quality control of meat was evaluated not only in comparison with benchtop NIR spectroscopy, but also in comparison with other analytical techniques, including optical spectroscopy (e.g., visible spectroscopy) [142,143].

### 4.4. Fish

Countering seafood mislabeling is receiving increased attention, as numerous cases of food fraud involving fish and seafood products were reported lately [148,149,150]. The scale of the problem can be well highlighted by economic-driven fraud reaching the entire fish supply chain, where on several occasions, a substitution for valuable fish with a cheaper species occurred [148]. Therefore, considerable research efforts have recently been directed at developing effective and reliable miniaturized NIR methods in this area of application. Miniaturized spectrometers were concluded to be feasible to provide accurate discrimination between fish species, as well as to quantitatively predict the main chemical contents in fish flesh; for example, fat composition, protein, or lipids (Table 6). 

To reflect state-of-the-art and currently undertaken research directions, Pennisi et al. [157] recently demonstrated highly accurate results using handheld NIR spectrometers for direct screening of a production line of cuttlefish and musky octopus. The authors emphasized the decisive practical gain from using miniaturized NIR instrumentation, with a greatly reduced complexity and the execution of the analysis that make it much more practical for successfully adoption in the challenging conditions commonly found at fish and seafood production sites.

### 4.5. Fruits and Vegetables

Fruits and vegetables are also among the most-studied items in the development of analytical miniaturized NIR spectroscopy, with equal interests in both the food and agriculture sectors. Most often, the aim of the analyses was directed towards the prediction of the quality parameters of fruits and vegetables (Table 7). Several canonical types of analysis were used; i.e., moisture level, protein content, and total sugar content (i.e., BRIX index), as well as the metrics of fruit maturity, content of soluble solid, titratable acidity and ascorbic acid, extractable polyphenols, etc. Often, more than one property of interest could be simultaneously predicted from a single spectrum. Importantly, in most cases, the full feasibility of miniaturized NIR spectrometers to perform those analyses directly under field conditions and/or during fruit ripening was demonstrated. Other than those, the qualitative assessments of relevant properties also included identification of the variety and/or geographical origin, assessment of the refrigerated-storage duration, and authenticity checks. Notably, there were studies demonstrating the potential for rapid discrimination of fraud due to the mislabeling of conventionally produced fruits as organic ones; e.g., in the case of pineapples [150]. A similar potential can be concluded for vegetable analysis, with the example of the examination of spinach leaves in situ, directly on the plant, in which a green color, the texture, and dry matter were detected using a miniaturized NIR instrument [158,159,160,161]. Such a capacity can be effectively used in the optimization process of cultural practices, such as fertilization and irrigation and to assess the quality of a vegetable when harvested.

### 4.6. Beverages and Syrups

Considering the success of miniaturized NIR spectroscopy in predicting critical qualitative and quantitative properties of foods that feature considerable complexity, such as meat, fresh fruits, or vegetables, one should expect that the analysis of beverages or syrups should be within reach of this technology as well. An inspection of the recent literature indicated that miniaturized NIR sensors can provide the rapid assessment of sugar content in such samples in a wide range of concentrations, and in a robust manner (Table 8). The prediction of other chemical contents, such as polyphenols, amino acids, caffeine, and theanine, as well as the quantitative assessment of the adulteration level, could be concluded in the available literature. 

Noteworthy classification examples that included discrimination between different beer brands or sake varieties using miniaturized NIR spectroscopy should be mentioned as well [179,180].

### 4.7. Miscellaneous

Other examples of recent food-analysis studies should also be noted that further demonstrated the versatility of miniaturized NIR spectrometers, in which they were successfully applied to qualitative and quantitative analyses in a variety of cases, from whole foods to highly processed products (Table 9). Content analysis included, e.g., carbohydrates, fats and oils, fiber, proteins, and sugar as well as general energetic/nutritional value. Specific chemical constituents could be successfully analyzed; for instance, piperine in black pepper, or insect proteins in fitness bars [73,99]. Detection of adulterants; for example, in palm oil [176], as well as the quantitative analysis of adulteration was feasible using miniaturized NIR spectroscopy. Examples of successful authentication or classification/identification of various food products included the different quality grades and geographical origins of rice, as well as of basic food powders such as sugar, salt, cream, flour, corn, rice, bean, and potato powders [177]. The prediction of other quality parameters of more general nature; e.g., egg storage time assessment, was permissible as well [174].

An inspection of the available literature well demonstrated the versatility and superior utility of miniaturized NIR spectrometers in the diverse realm of food analysis and quality control. On the other hand, attention should be also given to some of the limitations in the applicability of handheld NIR instruments; for example, chlorophyll content could not be accurately predicted in canola seed, as determined by Barthet et al. [186].

## 5. Current Trends in Method Development

### 5.1. Systematic Evaluation of Calibration Methods

In parallel to their revolutionary practical advantages, miniaturized technologies also impose inevitable limitations on the optical/spectral capabilities of the very compact NIR instruments. Spectra measured in narrow wavenumber regions with a relatively low resolution and an often-inferior S/N ratio, as compared with benchtop spectrometers, place a particular need on performing systematic feasibility studies of miniaturized NIR spectrometers in given analytical scenarios. In this case, the evaluation of numerous spectral pretreatments and calibration algorithms, including artificial neural networks (ANNs) and nonlinear regression methods (e.g., Gaussian process regression (GPR)) is recommended to establish the best data-analytical approach for a given sensor in a specific analytical scenario. As demonstrated, this may result in a sizeable improvement in the predictive performance of portable NIR spectrometers, surpassing PLSR with minimal or no penalties to the prediction performance of handheld vs. benchtop spectroscopy in difficult analyses (e.g., moisture analysis in a plant matrix). Advanced calibration enables miniaturized spectrometers to nearly match the performance of benchtop instruments.

As shown by Mayr et al. [31], the analytical performance of miniaturized spectrometers in a challenging scenario of the quantitative analysis of the moisture in chemically complex plant matrices could be significantly improved by carefully evaluating several different spectral pretreatments and calibration methods, which were systematically evaluated for each of the considered benchtop and miniaturized NIR spectrometers. In that study, two benchtop instruments (NIRFlex N-500 and MPA I) and three miniaturized instruments (microPHAZIR, MicroNIR 2200, and MicroNIR 1700 ES) were used, while the analysis of the moisture content was performed for 192 samples of dried plant extracts, consisting of five different plants of different geographical origins harvested at different times over two years. The samples included extracts laced with a drying agent, as well as unpretreated samples that presented a less-stable matrix that was prone to variations in the moisture content. The reference moisture analysis for calibration was performed using an industry standard; i.e., the Karl Fischer titration method. Moisture content is one of the most important quality parameters of numerous food products, including spices, teas, fruits, and vegetables, as well as in herbal medicines. It is decisive for the product stability and shelf life, and requires close monitoring. In the analyzed scenario, for the calibration/prediction procedure, in addition to the standard PLSR method, GPR and ANN models also were constructed for the spectral sets of each instrument. For each spectral set, a systematic evaluation of the best pretreatment combination was conducted as well. The prediction performances of those calibration models were evaluated through the root-mean-square error of prediction (RMSEP) determined for an independent test set (Table 10). The nonlinear GPR and ANN methods were noted to offer substantially improved performances in the case of poorer quality of the spectra from certain instruments, as well as in the case of the more challenging analysis of unpretreated samples. In this case, the miniaturized spectrometers offered a prediction performance at the level of the benchtop instruments (Table 10). Moreover, the samples in their native states proved to be more difficult to analyze for all evaluated instruments when using PLSR calibration [31]. That study demonstrated the potential of improving the analytical figure of merit of micro-NIR analysis for less-than-ideal data-sets; e.g., resulting from the difficult nature of the analyzed sample (i.e., chemically complex plant matrix) and the reduced quality of the spectra (i.e., narrow spectral region, lower resolution, and poorer S/N ratio typically accepted for the miniaturized spectrometers). This suggested that through systematic evaluation and selection of the data-analytical scheme, the penalty to the accuracy resulting from the hardware miniaturization can be compensated by using GPR or ANN calibration. 

### 5.2. NIR Sensor Fusion

Data-fusion methodologies offer considerable potential, effectively combining strengths of different analytical techniques. The opportunities stemming from this concept for food and beverage authentication and quality assessment were discussed in detail by Borràs et al. [196]. However, relatively greater attention was paid to using conventional benchtop NIR spectroscopy as the component of the fused approaches.

For example, an integrated analytical framework using fused physicochemical analyses, benchtop NIR spectroscopy, and melissopalynology (pH, electrical conductivity, and humidity) was recently presented by Bodor et al. [197]. An analytical method was developed to check the authenticity of Hungarian honey, with PCA-LDA models built to classify the different botanical and geographic sources using individual and fused data at a low level (i.e., low-level data fusion). Optimization of the number of principal components (PCs) and external validation were applied to all models. The botanical origin classification models demonstrated >90% and >55% accuracy levels for the melissopalynology and NIR methods when used separately. Improved results were obtained by combining physicochemical, melissopalynology, and NIR techniques, resulting in >99% and >81% accuracy of the botanical and geographic origin classification models developed for the fused data, respectively. Although a benchtop NIR spectrometer was used in that study, a similar method can likely be designed to include miniaturized/portable NIR instruments as well.

On the other hand, a direct improvement of the performance of NIR sensors with mutually exclusive operational spectral regions can be accomplished by data fusion as well. Several cost-effective miniaturized NIR sensors appeared on the market that were specifically intended for food analysis [5,6,7,77]. These devices are potentially very interesting for the small-scale manufacturing of food, which is becoming increasingly popular. Efficient quality control in such production poses considerable difficulties, and the final product quality depends on the supplied ingredients. Miniaturized NIR spectrometers are particularly promising in such scenarios, but often manifest inferior performances compared to benchtop NIR spectrometers; in certain cases, no successful calibration by PLSR could be obtained for some sensors in particularly challenging analyses [31,99]. As discussed in the previous section, advanced calibration methods offer promising potential in such cases. Further gains can be obtained with sensor fusion, which offers a convenient uplift in performance by combining spectra measured in different wavenumber regions for extended access to chemical information for more reliable calibration.

Although still relatively scarce, studies of effective data-fusion strategies that integrated miniaturized NIR spectrometers have begun to attract growing attention in the fields of food and agriculture. In the study of Bec et al. [73], a benchtop (Buchi NIRFlex N-500) and three miniaturized (MicroNIR 1700 ES, Tellspec Enterprise sensor, and SCiO sensor) NIR spectrometers were evaluated and optimized within a calibration framework based on the PLSR and GPR methods for prediction of the protein content in fitness bars containing edible insect material. The analyzed protein content in the calibration series was between 19.3 and 23.0% (Table 11). In the nondestructive analysis of intact bars, the RMSEP values from the PLSR prediction were determined to be 0.611% for the benchtop, and remained in the range of 0.545 to 0.659% for the miniaturized spectrometers. The predictions by GPR models were 0.506% (benchtop) and 0.482–0.580% (miniaturized). When taking into account the milled samples, the corresponding RMSEP values for the PLSR prediction were improved to 0.210% for the benchtop spectrometer, but remained in the inferior range of 0.525–0.571% for the miniaturized devices. However, the RMSEP values for GPR prediction for the miniaturized spectrometers were noticeably improved to 0.230% (MicroNIR 1700 ES), 0.326% (Tellspec), and 0.338% (SCiO). In combination with the PLSR method, the portable instruments showed a significantly lower predictive performance as measured by the RMSEP values determined for the data from an independent test set. Using a nonlinear GPR calibration method significantly improved the accuracy of prediction of the miniaturized spectrometers, with the MicroNIR achieving a performance equal to the stationary instrument, while only a slightly worse performance was achieved by the Tellspec and SCiO sensors.

**Figure 8:**
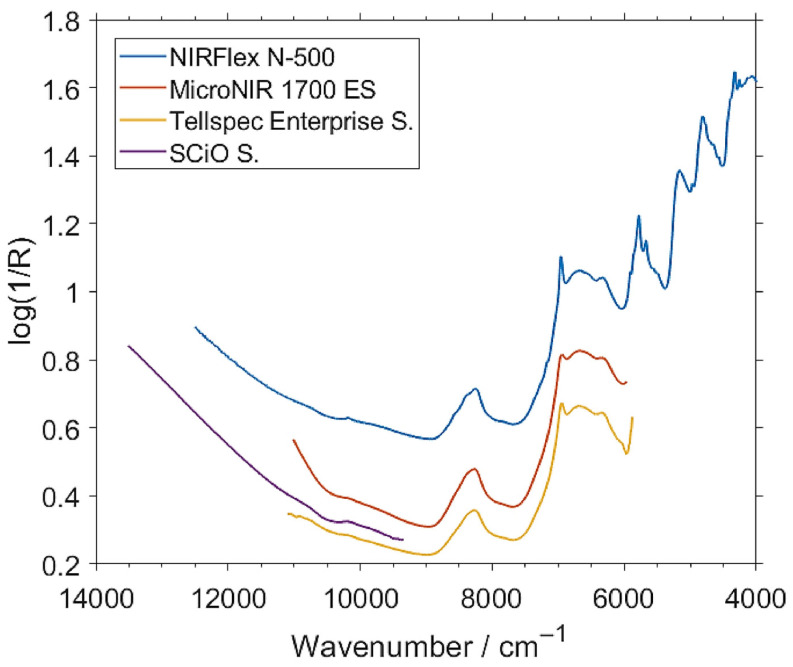
Unpretreated NIR spectra of exemplary intact insect protein fitness bar samples measured by a benchtop NIRFlex N-500 and three miniaturized (MicroNIR 1700 ES, Tellspec Enterprise sensor, and SCiO sensor) spectrometers. The two latter sensors are cost-effective designs specifically intended for food analysis by consumers. Reproduced (CC-BY 4.0 license) from [73].

A further increase in the reliability of the analysis can be achieved by low-level fusion of the spectra from spectrometers measuring largely different, only partially overlapping fragments of NIR spectrum, such as the SCiO and Tellspec instruments considered in that study (Figure 8). The Tellspec and SCiO sensors cover highly complementary VIS/SW-NIR and NIR wavelength ranges, with only a narrow overlap of the spectral regions measured by these two devices (Figure 8). Therefore, an attempt was made to aggregate (i.e., concatenate) the data from these sensors to effectively provide the calibration model with an extended spectral region compared to any of these two spectrometers used separately. In the GPR calibration and test-set validation performed on the fused data (Tellspec + SCiO), the RMSEP values were improved to 0.517% (for intact samples) and 0.295% (for milled samples). Because the Tellspec and SCiO sensors are consumer-oriented devices with superior affordability, it is still a cost-effective and economical option to use them together to increase the accuracy and reliability of an analysis. Fused-sensor NIR spectroscopy, with the combined strengths of multiple miniaturized portable instruments, can be successfully used for rapid, nondestructive analysis of total protein content with better performance than the sensors used separately.

Furthermore, Cavallini et al. [198] recently explored a midlevel data-fusion approach for three different NIR spectrometers: SCiO, MicroNIR, and a benchtop instrument (Bruker MPA). Additionally, covariance selection (CovSel) [199] and common dimensions (ComDim) [200] approaches were applied as well to establish a robust approach for distinguishing between fresh and frozen cephalopods. Interestingly, the authors noted that the similarities and differences among the datasets measured by these three instruments reflected their design characteristics. 

The conclusions that might be drawn from the data-fusion studies involving miniaturized NIR sensors suggest that the profound instrumental differences often observed for these devices create a particular opportunity for fusion approaches to provide direct gains in analytical performance. 

On the other hand, a different approach to reinforcing an analytical framework using miniaturized NIR spectrometers through a data-fusion approach stems from parallelization of spectra pretreatments. As concluded in numerous case studies, and as outlined in other sections of this review, miniaturized sensors often provide spectra of inferior quality, in various terms, compared to full-scale benchtop instruments. Often, most of the resulting distortions of the spectra (e.g., scattering profile, noise) can be corrected or suppressed by separately applied algorithms. However, a promising alternative is provided by applying the pretreatments in parallel and fusing the resulting datasets for further calibration and prediction. The methodological background of this concept was recently exhaustively presented by Mishra et al. [201]. Given the importance of the pretreatment step in processing the challenging spectral datasets often encountered in agri-food NIR analysis using miniaturized spectrometers, it should be expected that parallelized spectra pretreatment via data fusion will attract increasing attention in the development of analytical methods in the reviewed area of application.

### 5.3. Chemical Interpretation of Calibration Models and Instrumental Differences

With the recently provided availability of in silico simulated NIR spectra, it has become feasible to elucidate in detail the sensor sensitivity to specific chemical information, and thus gain a deeper insight into the critical factors that affect its prediction performance in a given analytical application. Accordingly, the differences in the prediction power of caffeine and L-theanine content in black tea using the microPHAZIR and MicroNIR instruments were analyzed in detail in a recent investigation by Mayr et al. [100]. The authors concluded that the sensitivities of these two miniaturized spectrometers to the characteristic absorption bands of these two components were distinct. This resulted from the observed substantial differences in the way the different NIR instruments recorded the chemical information of caffeine compared to theanine. Handheld spectrometers have shown a limited suitability for assessing theanine, as the meaningful absorption of this constituent falls outside the spectral region measured by these devices. On the contrary, the most characteristic absorption of caffeine is acquired by these handhelds, and as a consequence, their performance in analyzing caffeine content is comparable to that of a benchtop device. These observations suggest that the application of both spectrometers in the analysis of compounds structurally similar to caffeine may be successful, while in the chemicals structurally similar to theanine, it may be challenging for both instruments [100]. Similar conclusions have been drawn from the combined interpretation of the simulated spectrum of piperine and the chemometric models calibrated for the prediction of that chemical in the plant matrix of black pepper from the spectral datasets acquired by the same set of miniaturized spectrometers [99]. The noticeably different spectral regions that these devices measured illustrated a clear distinction between their abilities to acquire the meaningful absorption of a given constituent; in that case, piperine [99].

**Figure 9:**
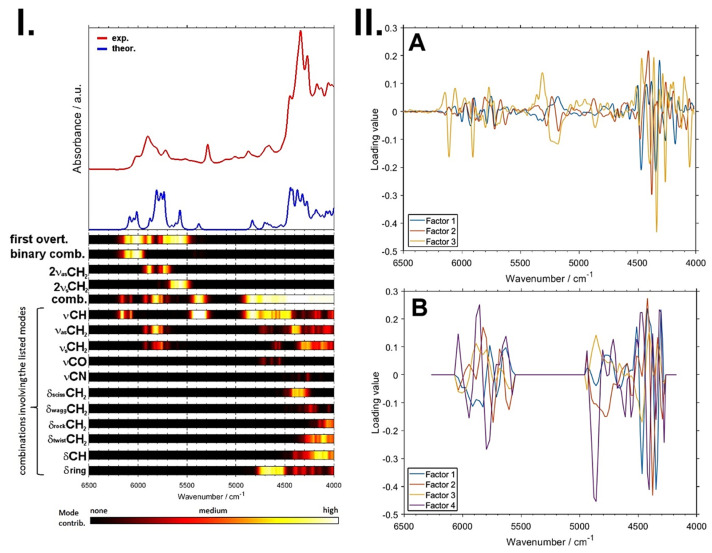
Panel (**I**): simulated spectrum elucidates vibrational contributions to NIR spectrum of piperine. Panel (**II**): this information enables the interpretation of the chemical information in the loading plots for the PLSR model of piperine content in black pepper developed for the NIR spectral sets measured with (**A**) a benchtop Büchi NIRFlex N-500; and (**B**) a miniaturized microPHAZIR spectrometer. Reproduced (CC-BY 4.0 license) from [97].

Another good example of how the comprehension of chemical information can be used in such scenario was provided by the further investigation by Grabska et al. into the patterns unveiled in the above-discussed studies [97]. In that study, the detailed band assignments of piperine offered insights into the correspondence of the PLS factors in the models describing piperine content in black pepper samples. The NIR vibrational contributions of piperine could be roughly established. The analysis using a benchtop spectrometer NIRFlex N-500 (Figure 9A) and a handheld microPHAZIR (Figure 9B) was dissected. The narrow spectral region in which the microPHAZIR operates (6266–4173 cm^−1^) was just enough to acquire the most meaningful absorption of piperine, with only weak second overtones and ternary combination bands populating the spectrum above 6150 cm^−1^. Firstly, as expected, the structure of the loading plots for all factors indicated a clear correspondence with the absorption features of piperine. Interestingly, exclusion of the region between 5550 and 4950 cm^−1^ improved the performance of the prediction for the microPHAZIR. Comparing this information with the determined vibrational assignments, one may conclude that the contributions from weak CH combination bands of piperine to the NIR spectrum of black pepper were not acquired to a satisfactory degree by the microPHAZIR. The model constructed for the microPHAZIR required four factors to obtain the maximum predictive performance, while for the dataset from the benchtop spectrometer, the optimal number of factors was three. The structures of the first factors for both cases are quite similar, and these seem to capture the most intense bands of piperine. Note, the structure of the third factor in the case of benchtop spectrometer clearly stands out from the remaining ones, e.g., above 6000 cm^−1^ and in the region of 5300–4900 cm^−1^. At these wavenumbers, one may see, standing out from the rest as well, the contributions from the combination bands involving CH and, to lesser extent, the ring deformation bands of piperine. The presence of the combinations involving ring deformation were also viable in the second factor for the benchtop spectrometer, where a distinct structure was observed near 4750–4500 cm^−1^.

The conclusions drawn in this study indicated that the laboratory spectrometer appeared to be more sensitive to the specific vibrations of piperine in black pepper. As shown, for example, by the factor 3 corresponding to νCH combination bands of piperine that was clearly distinct from the others (i.e., F-1 and F-2). There was also a significant part of the ring-deformation bands that contributed to the second and third factors in the PLSR model calibrated for the spectral set measured by the benchtop NIRFlex N-500 spectrometer. Therefore, in this case, a more accurate association between the distinct chemical information and the particular factors in the regression model was recorded.

In contrast, the factors in the PLSR model constructed for the spectral set from the microPHAZIR appeared to be less specific to individual vibrations of piperine. Consequently, the microPHAZIR was less capable of following fine spectral variations representing intensity changes in the spectrum related to a specific chemical constituent.

It is likely that the poorer spectral resolution of the miniaturized spectrometer also played a role here. It remains to be seen whether this observation can explain, at least in part, the poorer performance of the microPHAZIR spectrometer compared to the laboratory spectrometer in the analysis under discussion (root-mean-square of prediction; i.e., RMSEP of 0.30 and 0.18% *w*/*w*, respectively) [97].

### 5.4. Calibration Transfer

A calibration model, regardless of whether the intended problem to solve concerns classification (e.g., food authentication, identification of variety, brand, origin, etc.) or quantification (i.e., prediction of concentration of a targeted constituent, age of product, etc.) is generally valid only if the spectra used for predicting the property of interest is measured by the same spectrometer by which the calibration set was obtained. The critical difference here results from the disparity between two or more instruments with respect to key optical performance parameters such as the operating spectral region, wavelength accuracy, dynamic range, distance between sample and optics, the photometric response of the detector, etc. These differences are very likely to invalidate the prediction model when used with a different instrument, thus necessitating time-consuming calibration and validation for each specific spectrometer. On the other hand, in practical routines, often more than one NIR instrument; e.g., benchtop and miniaturized, or several miniaturized ones, are available at the site. Considerable gains in the efficiency of the analytical framework can be provided by the ability to perform calibration procedure on just one master instrument, which is then subsequently used for predictions from the spectra collected by the other sensors.

Various mathematical algorithms known as calibration-transfer or standardization strategies help convert models or data measured by a particular instrument for use by other instruments [202]. For this purpose, the following frameworks are widely used for eliminating the need for full recalibration: standardization of model coefficients, spectral responses, direct standardization (DS), segmental direct standardization (PDS), and spectral variations at each wavelength [202].

Examples demonstrating the utility value of calibration transfer in food analysis and quality control appeared in literature shortly after the first miniaturized spectrometers were popularized on the market. For example, the aim of the study of Pierna et al. [203] was to assess the possibility of transferring the calibration from the benchtop Foss NIRSystem 6500 instrument to the miniaturized microPHAZIR. In that case, good calibration models were obtained for the various feed properties (fat, fiber, protein, and starch) that were developed on the Foss NIRSystem 6500 on the basis of the spectral database of 9164 samples, and these were subsequently successfully transferred to the handheld spectrometer.

In another example, Zamora-Rojaset et al. [142] evaluated the performance of a miniaturized handheld NIRS instrument for assembling a meat-quality database from a high-performance benchtop NIR spectrometer. The authors showed that large databases of spectra of important samples collected over several years could be successfully transferred to a miniaturized, handheld NIR instrument, which is possibly better suited for in situ analysis on an industrial scale. The successful transfer of the database to the miniaturized NIR instrument provided considerable gains in efficiency and throughput of the analysis, particularly in a large-scale, low-cost online in situ analysis, a scenario that is commonly found in food industries.

## 6. Summary of Current Trends and Future Prospects

NIR spectroscopy is a powerful tool for qualitative and quantitative analyses involving different types of samples used in various industries. The canonical advantages of NIR spectroscopy; i.e., nondestructive analysis, high-throughput capacity, cost-effectiveness, and its “green” nature (i.e., no chemical solvent used, no expenditure of the product for the needs of analysis), form a perfect synergy with the novel, miniaturized sensor technology. With the advent of portable spectrometers, decisive enhancement of NIR spectroscopy in its utility in the agri-food sector was offered. The unique value of miniaturized NIR spectroscopy is best manifested in this sector, on which the analysis of complex, chemically and physically diverse samples of natural origin is focused; i.e., raw food materials, whole and highly processed foods, and agricultural crops. Importantly, this technology permits the persistent monitoring of food quality at every stage of its complex production and supply chain, from the farm to the shelf. In these applications, the revolutionary advantage of miniaturized sensors is manifested by the capacity to perform rapid, high-throughput analyses with no need for sample preparation directly onsite.

Accompanying progress in technology, recent breakthroughs in spectral-analysis methods and fundamental science have decisively changed our understanding of NIR spectra and largely expanded the potential for new applications. Comprehension of chemical information enables the interpretation of calibration models, while sensor fusion enhances the availability of the information correlated with food-quality parameters. The progress in data-analytical methods and the fundamental science underlying NIR spectra permit the knowledge-based design and optimization of the analytical application of NIR spectroscopy. These qualities have directly translated to a thriving development of new applications of miniaturized NIR spectroscopy, as it is readily available to combat food-safety risks resulting from the globalization of the food market. Hence, it has become widely adopted in this critically important sector of public interest.

While immensely successful and widespread, the technology and applications of miniaturized NIR spectroscopy still face certain challenges that should be considered in the future. Unexplored areas such as sensor deterioration and transferability of the models to new generations of instruments have been signaled by recent reviews published in the field. On the other hand, ultraminiaturization and the trend aiming at providing ordinary consumers with a spectrometer in their pocket urge the reinforced reliability of precalibrated models, as well as the user-friendliness and accessibility of cloud-based services that are intended for the processing and analysis of the spectra from such sensors. The reliable and fail-proof operation of NIR spectrometers integrated with smartphones by nonexpert personnel and ordinary consumers would mark the next cornerstone of this technology in food analysis, provided that the outlined challenges are successfully addressed.

## Figures and Tables

**Table 1:** The operational characteristics of selected miniaturized NIR spectrometers available on the market in comparison with two exemplary benchtop FT-NIR devices.

	Spectrom. (Vendor)		Key Components		Operational Wavelength Region		Optical Performance		Control/ Data Transfer/ Power Delivery	Weight (g)
		Src.	Wavelength Selector	Detector	(nm)	(cm^−1^)	Resolution (at l) ^(a)^ (nm)	S/N		
**Benchtop**	NIRFlex N-500 (Büchi)	TH (×2)	Polarization interferometer (FT)	InGaAs (TE-cooled)	800–2500	12,500–4000	Avg. 1	10,000:1	PC/ LAN/ 230 V	15,000
	Spectrum Two (PerkinElmer)	TH	Michelson interferometer (FT)	InGaAs (air-cooled)	680–4800	14,700–3800	0.8–6.4 (at 1000)	N/A	PC/ LAN or USB/ 230 V	13,000
**Miniaturized**	microPHAZIR (Thermo Fisher Scientific)	TH	MOEMS Hadamard mask (HT)	InGaAs	1596–2396	6267–4173	11	N/A	Autonomous/ USB/ Li-ion cell	1250
	MicroNIR 1700 ES (VIAVI)	TH (×2)	LVF	InGaAs (array; 128 elements)	908–1676	11,013–5967	12.5 (at 1000) 25 (at 2000)	23,000:1	PC/ USB/ USB	58
	SCiO (Consumer Physics)	LED	Bandpass filter	Si photodiode (array, 12 elements)	740–1070	13,514–9346	N/A ^(b)^	N/A	Smartphone (Bluetooth)/ cloud/ Li-ion cell	35
	NIR-S-G1 (InnoSpectra)	TH (×2)	stationary dispersive grating and MOEMS DMD	InGaAs	900–1700	11,111–5882	10	6000:1	Smartphone (Bluetooth)/ cloud/ Li-ion cell	136
	NIRONE Sensor S (Spectral Engines)	TH (×2)	MOEMS Fabry–Pérot interferometer	S1.4: InGaAs S1.7–S2.5: ‘extended’ InGaAs	1100–1350 (S1.4) 1350–1650 (S1.7) 1550–1950 (S2.0) 1750–2150 (S2.2) 2000–2450 (S2.5)	9090–7407 7407–6060 6451–5128 5714–4651 5000–4081	12–16 13–17 15–21 16–22 18–28	15,000:1–1500:1 (S1.4–S2.5)	Smartphone (Bluetooth)/ cloud/ Li-ion cell	15
	NeoSpectra-Scanner (Si-Ware Systems)	TH	MEMS Michelson interferometer (FT)	InGaAs	1350–2500	7407–4000	16 (at 1550)	N/A	Smartphone (Bluetooth)/ cloud/ Li-ion cell	1000
	nanoFTIR NIR (SouthNest Technology)	TH	Michelson interferometer (large mirror; FT)	InGaAs	800–2600	12,500–3846	2.5 (at 1000) 6 (at 1600) 13 (at 2400)	9000:1	PC/ USB/ USB	220
	LabSpec4 (ASD Inc., Yokohama, Japan)	TH	Dispersive (reflective holographic diffraction grating)	3 detectors: Vis-NIR (350–1000 nm): Si (array, 512 elements) SW-NIR: InGaAs (1001–1800 nm) and (1801–2500 nm) TE-cooled	350–2500	28,571–4000	3 (at 700) 10 (at 1400/2100)	9000:1 (700 nm) 9000:1 (1400 nm) 4000:1 (2100 nm)	PC/USB /acid-gel battery or 230 V	5440

^(a)^ “At wavelength” parameter listed if available in the data sheet provided by the vendor; ^(b)^ SCiO presents interpolated spectra to the operator with 1 nm data spacing, but the real resolution is considerably lower. Abbreviations: Src.—source; TH—tungsten halogen; TE—thermo-electric; ×2—duplicated element; N/A—not available. Unless stated otherwise, single-element detectors are used in the listed instruments. Multiple entries listed for some instruments correspond to different variants of the same model with specific factory settings.

**Table 2:** Approximate positions of NIR bands of the major classes of chemical compounds commonly present in foodstuffs (excluding water). Reproduced (CC-BY 4.0 license) from [29].

Wavenumber in cm^−1^	Wavelength in nm	Vibrational Mode Assignment and the Associated Most Characteristic Compounds ^(a)^
8250	1210	3 C–H str. (C-H rich compounds; e.g., carbohydrates, lipids)
7375–7150	1355–1400	2 C–H str. + C–H def. (carbohydrates, lipids)
6980	1435	2 N–H str. (proteins)
6750	1480	2 O–H str. (carbohydrates, alcohols, polyphenols)
6660	1500	2 N–H str. (proteins)
6500	1540	2 O–H str. (carbohydrates, alcohols, polyphenols upon matrix effects; e.g., hydrogen-bonded OH groups)
6400	1565	2 N–H str. (proteins)
6200–5800	1610–1725	2 C–H str. (carbohydrates, lipids)
5625	1780	2 C–H str. (C-H rich compounds; e.g., carbohydrates, lipids)
5500	1820	O–H str. + 2 C–O str. (carbohydrates)
5120	1955	3 C–O str. (carbohydrates)
4880	2050	N–H sym. str. + amide II (proteins)
4825	2075	O–H str. + O–H def. (alcohols, polyphenols)
4645	2155	Amide I + amide III (proteins)
4440	2255	O–H str. + O–H def. (carbohydrates, alcohols, polyphenols)
4360	2295	N–H str. + CO str. (proteins)

^(a)^ The numbers 2 and 3 denote the first and second overtones, respectively; a plus sign (+) denotes combination bands.

**Table 3:** Recent research activity oriented toward miniaturized NIR spectroscopy for analysis of milk.

Ref.	Scope	Sample	Miniaturized NIR Instr.	Data-Analytical Framework ^(a)^	General Remarks (Applicability/ Performance)
[107]	Performance evaluation of portable NIR spectrometer in authentication of organic milk	87 samples (full-fat, pasteurized retail milk) including 7 organic retail milks and 50 nonorganic retail milks	MicroNIR 1700	PCA, PLS-DA	Accurate discrimination between organic and conventional milk; less-successful class assignment of pasture milk samples; however, in both cases MicroNIR was noninferior to the benchtop NIR spectrometer
[108]	Goat milk authentication/detection of adulteration by cow milk	200 samples (54 pure goat milk samples and 146 adulterated samples)	NIRscan Nano	OC-PLS, PLS-DA, iSPA-PLS-DA	Miniaturized spectrometer successfully determined the authenticity of goat milk (adulteration with cow milk as risk scenario); all pure goat milk samples were correctly identified, with one adulterated sample misclassified in the test-set validation
[109]	Authentication of organic milk from other types of milk	37 organic retail milks and 50 nonorganic retail milks	SCiO	PLS-DA	Miniaturized NIR spectrometer was successful in distinguishing organic milk from conventional milk
[110]	Method development for handheld NIR spectrometer to collect raw milk spectra; analysis of protein, fat, and solids-nonfat (SNF) of raw milk; transfer of calibration models to another portable unit	542 fresh milk samples	microPHAZIR	MPLS	Successful calibration transfer demonstrated; sharing calibration models among several units indicated as essential for implementation of portable instruments for in situ analysis to provide indicators of milk composition at the farm level
[111]	Classification of milk samples according to their quality for improved monitoring in dairy facilities	903 fresh cow milk samples	microPHAZIR	PLSR, ANN	Miniaturized NIR spectroscopy provided considerable advantages at the milking stage using real-time monitoring of the quality-control parameters for each cow milk sample individually
[112]	Evaluation the capabilities of two portable NIR instruments (SCiO and NeoSpectra) in rapid, simple, and low-cost quantitative determination of macronutrients in commercial milk	45 commercial milks	SCiO, NeoSpectra	PCA, PLSR	Both SCiO and NeoSpectra could provide a fast and reliable analysis of fats in commercial milk; correct classification of milk according to fat level feasible; SCiO able to predict protein content and detect the presence or absence of lactose
[113]	Discrimination between regular and lactose-free ultrahigh-temperature (UHT) milks using benchtop FT-NIR and miniaturized NIR spectrometers, aiming at in-field analysis	71 samples; 41 lactose-free UHT milk and 30 regular UHT milk	MicroNIR 1700	PLS-DA, GA-LDA, SPA-LDA	Miniaturized NIR spectroscopy deemed feasible in discrimination between regular and lactose-free milk directly in the field
[114]	Development of miniaturized NIRS method for quick and simple on-site monitoring of the fatty-acid profile in raw milk at the farm level	108 raw milk samples	microPHAZIR	PLSR	Accurate classification of milk by miniaturized NIR spectroscopy at the farm level by fatty-acid-composition labeling; successful quantification of fatty-acid sums and healthy indices in individual cow’s milk; prediction of individual fatty acids, and saturated fatty acids in particular, deemed feasible as well
[115]	Development of NIR analytical method for onsite, contactless monitoring of milk quality	17 milk specimens (commercially available in Italian markets)	MicroNIR OnSite	PCA, PLSR	Accurate differentiation of milk as a function of the distribution of fatty acids in a rapid and nondestructive manner using the MicroNIR spectrometer

^(a)^ Abbreviations: ANN—artificial neural network; GA-LDA—genetic algorithm linear discriminant analysis; MPLS—modified partial least squares; OC-PLS—one-class partial least squares; PCA—principal component analysis; PLSR—partial-least-squares regression; PLS-DA—partial-least-squares discriminant analysis; iSPA-PLS-DA—successive projections algorithm for interval selection in PLS-DA; SPA-LDA—successive projection algorithm–linear discriminant analysis.

**Table 4:** Recent research activity oriented toward miniaturized NIR spectroscopy in the area of dairy-product applications.

Ref.	Scope	Sample	Miniaturized NIR Instr.	Data-Analytical Framework ^(a)^	General Remarks (Applicability/ Performance)
[118]	Performance comparison of benchtop vs. miniaturized NIR spectrometer in determining quality parameters of cheese	46 cheese samples (20 of hard cheese, 26 samples of semihard cheese, respective to water content)	SCiO	PLSR	Good accuracy of a miniaturized, extremely cost-effective NIR spectrometer in analyzing quality parameters of cheese; acceptable performance even when using consumer-aimed software
[119]	Three different NIR instruments for online determination of fat and dry matter in cheese blocks	160 cheeses from 10 production batches	MicroNIR 1700	PLSR	Miniaturized NIR spectroscopy enables improved control of the cheese-making process through early detection of the deviations from the target quality during the production process
[120]	Development and validation of rapid quantification technique for intact casein and total protein in cheddar cheese	49 white and yellow cheddar cheese samples	SCiO	PLSR, iPLSR	Successful quantification of intact casein and total protein in cheddar cheese by a miniaturized, ultra-cost-effective NIR spectrometer; method can be implemented in manufacturing facilities as a low-cost quality-control tool for cheddar cheese and processed cheese
[121]	To develop a rapid analytical method for determination of the content of key cheese quality and ripening indicator compounds; vibrational spectroscopic characterization of biochemical changes occurring during the ripening process of cheese	36 white cheese cubes were produced in 2 batches, forming 72 cubes for analysis, and each of them weighed approximately 400 g	NeoSpectra	PLSR	Handheld NIR spectrometer deemed suitable for rapid, simple, in situ monitoring of the quality of cheese during aging; real-time monitoring of the deviations in the manufacturing process indicated as feasible
[122]	Feasibility study for a low-cost NIR spectrometer to predict total nitrogen, soluble nitrogen, ripening index, major minerals, and fatty acids in cheese	104 ground cheese samples	SCiO	MPLS	Miniaturized, ultra-cost-effective NIR spectrometer provided an accuracy in the prediction of the targeted traits similar to benchtop devices
[123]	Prediction of dry matter, fat, fat/dry matter, proteins, and proteins/dry matter in Grana Padano cheese; feasibility study for screening operations of production batches in the fire-branding step, in warehouses and at the packaging step, on cheese paste	195 samples of Grana Padano	XNIRTM (Dinamica Generale)	PLSR	Portable NIR spectrometer demonstrated satisfactory predictive performance of the chemical composition of Grana Padano cheese, with performance metrics comparable to a benchtop FT-NIR instrument
[124]	Prediction of chemical contents (5 traits), pH, texture (2 traits), and color (5 traits) of 37 categories of cheese; comparison of 3 NIR instruments (2 benchtop) in reflectance and transmittance mode; different wavelength intervals	1050 different cheeses from 104 cheese factories	LabSpec2500 (ASD Inc.)	PLSR	The predictive performance of the visible/NIR portable spectrometer operating in diffuse reflectance mode was indicated as generally better than the 2 laboratory NIR instruments, both when the entire spectrum or selected intervals were considered and with the reflectance and transmittance modes examined; the portable instrument was suitable for analyzing the chemical composition of cheese in real time, without the need for sample uptake and processing
[125]	Rapid analysis of main compounds in milk powder	350 milk powders	FieldSpec Pro FR (ASD Inc.)	LS-SVM, PLSR	Handheld SW-NIR spectrometer was determined to be an excellent detector for the milk powder analysis, suiting the needs of industrial application
[126]	Feasibility study for visible and NIR spectroscopy to perform quantitative detection of the irradiation dose (0–6.0 kGy) in milk powder; irradiation by ^60^Co γ-rays	150 samples of milk powder	FieldSpec (ASD Inc.)	RC, PLSR, LS-SVM	Miniaturized NIR instrument fully suitable for performing the rapid online detection of irradiation doses of milk powder in a food-safety-monitoring scenario
[127]	differentiation of pure vs. adulterated milk powder	35 milk powder samples	microPHAZIR	PCA	Miniaturized NIR spectrometer was determined to be successful in the differentiation of pure vs. adulterated milk powder; the specificity of the nontargeted method was dependent on the type of adulterant; the use of complementary techniques (e.g., Raman spectroscopy) should be investigated to fully cover the adulterant classes

^(a)^ Abbreviations: iPLSR—interval partial-least-squares regression; MPLS—modified partial least squares; PCA—principal component analysis; PLSR—partial-least-squares regression; LS-SVM—least-squares support vector machine; RC—regression coefficients.

**Table 5:** Recent research activity oriented toward miniaturized NIR spectroscopy in the area of meat application.

Ref.	Scope	Sample	Miniaturized NIR Instr.	Data-Analytical Framework ^(a)^	General Remarks (Applicability/ Performance)
[129]	Performance comparison of benchtop vs. miniaturized NIR spectrometer in detecting meat fraud	63 samples of different meat types (beef: 9, chicken: 10, mutton: 10, turkey: 10, pork: 10, horse meat: 14)	microPHAZIR	PCA, PLSR	High-level meat adulterations (>10%): fully feasible with benchtop spectroscopy, improvements required for miniaturized instrument (e.g., larger sample set); low-level meat adulterations (<10%): improvements were needed for both types of instrumentation
[130]	Performance assessment of a miniaturized NIR spectrometer (NIRscan) for prediction of intramuscular fat in comparison with two portable and one visible/SW-NIR spectrometers	Lamb meat: frozen (609 samples), fresh (60 samples)	Labspec5000 Trek (ASD Inc.), LabSpec4, NIRscan Nano	PLSR, VIP	Prediction performance not affected by sample temperature-equilibration time; frozen samples: good performance of LabSpec5000, LabSpec4, and Trek instruments; bias (measurement timewise) observed for NIRscan Nano (instrumental variations); fresh meat: NIRscan Nano performed well and was a good alternative to other benchtop and handheld spectrophotometers for rapid and real-time classification of fresh lamb meat
[131]	Authentication of chicken meat by miniaturized NIR spectrometer	153 fresh chicken fillet samples	MicroNIR 1700	PLS-DA, CP-ANN, SVM, RSDE	Miniaturized NIR spectroscopy provided cost-efficient, rapid (<20 s for complete analysis), and reliable tool for monitoring meat authenticity (and quality) directly in the field
[132]	Feasibility study and method development for miniaturized NIR spectroscopy used as onsite tool for analyzing microbiological status of pork meat	252 samples of pork meat slices	microPHAZIR	PCA, PLSR, MPLS	Miniaturized NIR spectroscopy feasible for onsite prediction of microbiological status of pork meat with good accuracy; modified packaging atmosphere had no influence on performance
[133]	Miniaturized NIR spectrometer evaluated as onsite analyzer of meat quality traits in Iberian pig	Samples of Longissimus dorsi muscles were collected from 524 carcasses of Iberian pigs from “Sánchez Romero Carvajal Jabugo S.A.”	MicroNIR 1700	MPLS	Good accuracy of the method based on a handheld NIR device in analyzing intact pork loins directly at the industrial plant
[134]	Self-developed portable and low-cost isible/NIR detection device for predicting total volatile basic nitrogen (TVB-N) content analysis and assessing pork meat freshness	58 pork samples with different freshness attributes	Self-developed LED-based portable visible/NIR spectrometer (400–1100 nm)	MLR, PLSR	Nondestructive detection of TVB-N content in pork meat using a cost-effective, custom-designed miniaturized NIR spectrometer; streamlined instrument design with simplified structure and increased cost-effectiveness was indicated as feasible for further development
[135]	Analysis of color and pH value in pork meat using a new self-developed portable and low-cost visible/NIR detection	42 pork samples with different attributes of freshness	Self-developed LED-based portable visible/NIR spectrometer (400–1100 nm)	MLR	Nondestructive detection of pork freshness attributes, including color parameters and pH value, with the cost-effective, custom-designed miniaturized low-cost visible/NIR spectrometer
[136]	Feasibility study for using miniaturized NIR spectroscopy at the point of need to estimate the freshness of various foods including: beef sirloin, beef eyeround, pork sirloin, bass, salmon, corvina, tomato, and watermelon	8 food items: meat (beef sirloin, beef eyeround, pork sirloin), fish (salmon, bass, corvina), vegetable (tomato), and fruit (watermelon)	SCiO	SVM	Miniaturized, ultra-cost-effective NIR spectrometer successful in classification of foods by the aging day and by the chemical/microbial indicators (i.e., thiobarbituric acid and volatile basic nitrogen and bacteria levels); high accuracy, concluded to be fully satisfactory for point-of-need freshness assessment of meat, fish, vegetables, and fruits
[137]	Feasibility study for using miniaturized NIR spectroscopy to predict chemical parameters, technological and quality traits, fatty acids, and minerals in intact Longissimus thoracis and Trapezius obtained from the ribs of Charolais cattle	40 rib cuts taken at the level of the 5th rib were collected from 40 Charolais beef cattle	SCiO	MPLS	Miniaturized, ultra-cost-effective NIR spectrometer feasible in the online prediction of targeted beef quality traits; eliminated the need for commercial cuts, sampling, carcass deterioration, or grinding, thus avoiding product expenditures
[138]	Classifying chicken parts (breasts, thighs, and drumstick) using a portable NIR spectrometer; analysis of physical and chemical attributes (pH and color features) and chemical composition (protein, fat, moisture, and ash)	137 chicken samples (52 breasts, 40 thighs, and 45 drumsticks) and 90 samples obtained by grinding the chicken parts (30 breasts, 30 thighs, and 30 drumsticks)	DLN NIRscan Nano	LDA, RF, SVM	Portable NIR spectroscopy achieved good accuracy of classification of chicken meat, in which identification of different parts of chicken in the processing line was accomplished; authentication of shelf samples in the market for processed products was equally feasible
[139]	Feasibility study for using miniaturized NIR spectroscopy to detect adulteration in ground meat	Cuts of cow, pig, and chicken breast (undisclosed number of samples)	MicroNIR 1700	PLSR, SVR	Portable NIR spectrometer showed satisfactory performance in the quantification of beef in ground meat blends (chicken/beef, pork/beef, and chicken/beef/pork)
[140]	Feasibility study for miniaturized NIR spectroscopy to discriminate between different muscle types within each species of selected game animals, and to classify species regardless of the muscle	42 animal (12 impala (Aepyceros melampus), 15 eland (Taurotragus oryx) and 15 ostrich (Struthio camelus)	MicroNIR OnSite	PCA, PLS-DA	Miniaturized NIR spectroscopy successfully authenticated game meat, specifically impala, eland, and ostrich; discrimination between species (regardless of the muscle type under examination) was less challenging than identification of different muscles within each species
[141]	Feasibility study for miniaturized NIR spectroscopy to combat deliberate adulteration or accidental contamination of a pure veal product with pork and pork fat in a case study of a regional sausage product	84 samples of pure veal sausage product (30 samples (6 subsamples for each adulteration level) with an adulterated fat part, 30 samples (6 subsamples for each adulteration level) with an adulterated meat part, and 12 samples as genuine reference samples with no adulteration)	microPHAZIR	PCA, SVM	Meat adulteration: successful detection of adulteration down to 10% level (calc. for meat part only in the total composition of sausage), and down to 20% with through-package (polymer, double layer) scanning; fat adulteration: successful detection down to 20% (fat part only; i.e., 2.8% of the alteration of the total sausage composition)
[142]	Transfer (benchtop to handheld NIR instrument) of quantitative models for prediction of fat, moisture, and protein composition in ground pork samples	342 Iberian pork-muscle samples	microPHAZIR	PDS, MPLS, SDW, DS	Successful transfer of quantitative models for the prediction of fat, moisture, and protein composition in ground pork samples from benchtop to handheld NIR instrument; eight standardization samples deemed sufficient for standardization purposes
[143]	Feasibility study for visible/NIR spectrometer to discriminate enhanced quality pork; spectra were collected using intact chops from pork carcasses	148 pork carcasses	LabSpec4	PLS2-DA, PLSR	Portable visible/NIR spectrometer could not differentiate pork samples based on preslaughter diet or postslaughter carcass-chilling process; however, it was possible to segregate enhanced quality pork according to production factors and postmortem strategies such as pig breed, moisture enhancing, and ageing period
[144]	Performance comparison of three NIR instruments differing in size and characteristics: a transportable visible/NIR, a portable NIR, and a handheld NIR in the prediction of beef characteristics	178 beef samples (Longissimus thoracis muscle)	MicroNIR Aurora NIR, LabSpec 2500	PLSR, LMS	For the targeted 13 parameters of beef quality, three portable NIR spectrometers presented similar accuracies in prediction defined via external validation, with the most compact instrument (MicroNIR) tending to be the most precise; data-redundancy problems resulting from wideness of the spectrum and the number of data points suggested as a meaningful technical factor that affected the analytical performance of the different instruments
[145]	Feasibility study of a miniaturized NIR spectrometer to rapidly assess pork freshness	80 samples with four groups of 20 (storage in 4 °C for 2, 4, and 8 days)	MicroNIR 2200	PLSR, MLR, SPA, RC	Good performance of the miniaturized NIR spectrometer; suitable for nondestructive monitoring of thiobarbituric-acid-reactive substances in minced pork
[146]	Development of a method for handheld NIR instrument to predict fatty-acid (FA) composition and iodine value (IV) of pig subcutaneous fat	357 pigs	LabSpec4	PLSR	Portable NIR spectrometer was concluded to be suitable for predicting pig fat quality; successful implementation of the miniaturized sensor technology in a research abattoir, with spectra collected directly on the carcass, which enabled carcass sorting based on fat composition or hardness for marketing purposes
[147]	Feasibility study of a miniaturized NIR spectrometer to perform classification of individual Iberian pig carcasses into the four official quality categories	763 samples of Iberian pigs	microPHAZIR	PCA, PLS2-DA	Portable NIR spectroscopy successful in supporting the control of official quality-category assignment in Iberian pig carcasses, in commercial abattoirs while using subcutaneous fat samples

^(a)^ Abbreviations: CP-ANN—counter-propagation artificial neural network; DS—direct standardization; MLR—multilinear regression; MPLS—modified partial least squares; PCA—principal component analysis; PDS—piecewise direct standardization; PLSR—partial-least-squares regression; PLS-DA—partial-least-squares discriminant analysis; PLS2-DA—polynomial order of 2 partial-least-squares discriminant analysis; PLSR—partial-least-squares regression; RC—regression coefficients; RF—random forest; RSDE—random subspace discriminant ensemble; LDA—linear discriminant analysis; LSM—least-squares means; SDW—spectral difference by wavelengths; SPA—successive projection algorithm; SVM—support vector machine; SVR—support vector machine regression; VIP—variable importance in projection.

**Table 6:** Recent research activity oriented toward miniaturized NIR spectroscopy in the area of fish analysis.

Ref.	Scope	Sample	Miniaturized NIR Instr.	Data-Analytical Framework ^(a)^	General Remarks (Applicability/ Performance)
[151]	Performance evaluation for a handheld NIR device (in comparison with a FT-NIR benchtop spectrometer) in distinguishing fillets and patties of Atlantic cod from those of haddock	170 fresh fillets of *Gadus morhua* (*n* = 80) and *Melanogrammus aeglefinus* (*n* = 90)	MicroNIR OnSite	LDA	Handheld NIR device formed a simple, cost-effective, and reliable alternative to benchtop spectrometers for authentication of fish fillets and patties; the method was suitable for application in the fight against commercial fraud, and also was extended to authentication of fish species in processed products
[152]	Investigations of whole fish and fish fillets with a miniaturized NIR spectrometer; discriminating between high-quality fish from inexpensive lower-quality substitutes	30 fresh fish (3 red mullet, 6 mullet, 3 winter cod, 7 cod, 6 samlet, 5 salmon trout)	MicroNIR 1700	PCA, SIMCA	SIMCA analysis of the spectra measured by MicroNIR on the skin or flesh of whole fish or fish fillets provided correct authentication of the fish sample
[153]	Pocket-sized NIR sensor used for species identification in fish fillets	150 fish samples (9 fish species: *Merluccius merluccius*, *Pollachius virens*, *Epinephelus costae*, *Gadus morhua*, *Pleuronectes platessa*, *Sebastes norvegicus*, *Scomber scombrus*, *Chelidonichthys lucerna*, and *Synaptura cadenati*)	SCiO	Pretreatment and analysis of spectra performed using the functions built into the proprietary cloud service	Fish species were correctly identified with a global accuracy of 93.97–96.58% (validated by a method based on genetic marker); the method was concluded to be a good screening approach to counter fish-species fraud
[154]	Prediction of fat content in frozen skipjack by portable and benchtop NIR spectrometers	60 skipjack *Euthynnus pelamis* samples	FT 20 (Fantec Research Institute, Kosai, Japan)	PLSR	Portable NIR succeeded in the rapid determination of fat content by scanning the abdominal part of the fish body; for both instruments, the accuracy (determined via the RPD value) was higher at the abdominal part than at the central part of the body; the portable instrument was superior in analyzing the abdominal part
[155]	Prediction of nutritional values (protein, lipids, and moisture), and discrimination between sources (farmed vs. wild fish) and conditions (fresh or defrosted fish)	805 fish samples (133 Alaskan pollock (*Gadus chalcogrammu*), 204 Atlantic cod (*G. morhua*), 22 European plaice (*Pleuronectes platessa*), 264 common sole (*Solea solea*), and 182 turbot (*Psetta maxima*)	Tellspec Enterprise Sensor	PCA, LDA, PLSR, RF, LR, SVM, XGB	Good to excellent performance of the Tellspec sensor in both the prediction of nutritional values (protein, lipids, and moisture) and in authenticating the source and condition of all the studied fish species
[156]	Onsite determination of the fatty-acid composition of industrial fish oils from fish byproducts	269 different mixtures of 8 fish oil samples	MicroNIR OnSite	PLSR	The miniaturized NIR spectrometer successfully determined fish oil fat composition onsite in a fast and nondestructive way; attractive alternative to inefficient conventional ways of analysis
[157]	Performance of three NIR instruments in identifying storage conditions of fish products	50 fresh specimens of cuttlefish (*Sepia officinalis*) and musky octopus (*Eledone spp.*)	MicroNIR 1700, SCiO	PCA, PLS-DA	Very good classification accuracy of the miniaturized sensors; great practical gains were emphasized in the specifics of direct application on the production line

^(a)^ Abbreviations: PCA—principal component analysis; PLS-DA—partial-least-squares discriminant analysis; PLSR—partial-least-squares regression; RF—random forest; LDA—linear discriminant analysis; LR—logistic regression; SVM—support vector machine; XGB—extreme gradient boosting.

**Table 7:** Recent research activity oriented toward miniaturized NIR spectroscopy in the area of fruit and vegetable applications.

Ref.	Scope	Sample	Miniaturized NIR Instr.	Data-Analytical Framework ^(a)^	General Remarks (Applicability/ Performance)
[158]	Method development for onsite analyses of apples; detection of total antioxidant capacity and total soluble solids content	92 apples of seven cultivars	microPHAZIR	PCA, PLSR	Successful prediction of the total sugar content of apples of different varieties and the concentration of polyphenolic compounds in the peel of the fruits in nondestructive onsite analysis
[159]	Prediction of external and internal quality parameters of strawberries at harvest and during postharvest refrigerated storage using a handheld NIR spectrometer	189 strawberry punnets	microPHAZIR	MPLS, PLS-DA	Accurate prediction of internal quality parameters in strawberries using a handheld NIR instrument; however, room for further improvements and method tailoring to variety classification was indicated
[160]	Performance evaluation of different regression algorithms for the prediction of major physical-quality parameters in all citrus fruits using a handheld NIR spectrometer	611 samples belonging to the genus Citrus: 378 oranges (*Citrus sinensis* L. cv. ‘Powell Summer Navel’) (191 harvested in 2010 and 187 in 2011) and 233 mandarins (*Citrus reticulata* Blanco cv. ‘Clemevilla’)	microPHAZIR	MPLS	Miniaturized NIR spectroscopy combined with large databases and local regression algorithms provided robust on-tree prediction of citrus fruit quality
[161]	Method development for a portable NIR spectrometer to perform simultaneous discrimination between organically produced pineapple fruits and conventionally produced ones (i.e., organic vs. inorganic); prediction of total soluble solids	90 intact pineapple fruits	SCiO	KNN, PCA, LDA, PLSR, MSC-PCA-LDA	Portable NIR spectrometer coupled with the appropriate chemometric tools was suitable for rapid nondestructive examination of pineapple quality; successful detection of pineapple fraud-mislabeling of conventionally produced fruits as organic ones
[162]	Feasibility study for using a miniaturized and benchtop NIR instrument to predict quality-related parameters (soluble solid content, firmness, variety and postharvest storage duration under refrigeration) in intact plums	264 plums (*Prunus salicina* L.) cv. ‘Black Diamond’, ‘Golden Globe’, ‘Golden Japan’, ‘Fortune’, ‘Friar’, and ‘Santa Rosa’	microPHAZIR, Perten DA-7000	MPLS, PLSR, PCR	Similar levels of accuracy for miniaturized and benchtop NIR for the measurements of soluble solid content, variety, and refrigerated-storage duration; the prediction model developed using the diode-array spectrophotometer provided better results for the prediction of firmness
[163]	Development of in-field nondestructive analysis of titratable acidity and ascorbic acid content in acerola fruit during ripening	117 acerola fruit	MicroNIR 1700	PLSR, SVM	Fully satisfactory prediction ability of thw MicroNIR instrument during in-field monitoring of chemical parameters of interest in acerola fruits
[164]	Feasibility study for a miniaturized NIR instrument to perform “in vineyard” screening of extractable polyphenols in red grape skins	400 grape samples	MicroNIR 1700	PCA, LDA, MPLS, DPLS	MicroNIR instrument was successfully used for “in vineyard” screening of extractable polyphenols in red grape skins; however, challenges identified as environmental and physiological conditions interfered with sorting the berries according to their extractable polyphenol contents
[165]	Performance evaluation of handheld visible/NIR spectrometer in rapid nondestructive moisture content analysis in mangoes during solar drying	240 mango samples	F750, Felix Instruments	PLSR	Handheld visible/NIR spectroscopy was found to be a robust and effective method for rapid nondestructive monitoring of moisture during solar drying of mangoes
[166]	Feasibility study for portable NIR spectroscopy to be used in-field to assess the water status in diverse varieties (grown under different environmental conditions) of grapevine	160 individual primary adult leaves (20 leaves per cultivar) of the mid-upper part of the shoot	microPHAZIR	PCA, MPLS	Nondestructive, onsite NIR spectroscopic analysis reliably assessed the grapevine water status under field conditions
[167]	Quantification of water, protein, and soluble sugar in mulberry leaves using a miniaturized NIR spectrometer	83 mulberry leaves	MicroNIR 1700	PLSR	Handheld NIR spectrometers combined with wavelength optimization could rapidly predict water content in fresh mulberry leaves and crude protein in dried mulberry leaves; however, predictive performance was identified for the prediction of soluble sugar in mulberry leaves
[168]	Authentication of fengdous and quantitative analysis of mulberry fruits using a miniaturized NIR spectrometer	434 mulberry fruits	MicroNIR 1700	GA, CARS, PLSR	Several successful qualitative and quantitative plant analytical case studies were demonstrated for the handheld NIR instrument; several nutritional parameters were successfully determined
[169]	Development of a nondestructive and in situ quality evaluation of spinach plants using a miniaturized NIR spectrometer; assessment of spinach suitability for different uses once harvested	128 samples of spinach plants	microPHAZIR	MPLS, PLS-DA	Capability of miniaturized NIR spectroscopy to monitor important safety and quality parameters during the production of spinach was demonstrated
[170]	In situ monitoring of quality parameters in intact spinach using a miniaturized NIR spectrometer	149 spinach plants (*Spinacia oleracea* L, cv. ‘Solomon’, ‘Novico’, ‘Meerkat’, and ‘Gorilla’),	microPHAZIR	MPLS	Miniaturized NIR spectroscopy could perform an analysis of green color, the texture, and dry matter in spinach leaves in situ, on the plant; predicted properties were applicable in optimization of the fertilization and irrigation strategies
[171]	Development of a method for assessing tomato quality attributes nondestructively using a miniaturized NIR spectrometer	319 fresh market tomato samples	NeoSpectra	PLSR	Handheld NIR spectrometer could simultaneously determine several quality attributes of different types of tomatoes in a practical and rapid manner
[172]	Feasibility study for using a using miniaturized NIR spectrometer to determine quality attributes of tomato fruits	300 tomato fruits of the San Marzano variety	MicroNIR 1700	PLSR	Miniaturized NIR spectroscopy was indicated as a very potent tool and a real-time, cost-efficient measure to maintain the quality of the product, as demonstrated in a case study of the San Marzano tomato

^(a)^ Abbreviations: CARS—competitive adaptive reweighted sampling; DPLS—discriminant partial-least-squares analysis; GA—genetic algorithm; KNN—k-nearest neighbors; MPLS—modified partial least squares; PCA—principal component analysis; PLS-DA—partial-least-squares discriminant analysis; PLSR—partial-least-squares regression; LDA—linear discriminant analysis; SVM—support vector machine; NIPALS—nonlinear iterative partial least squares.

**Table 8:** Recent research activity oriented toward miniaturized NIR spectroscopy in the area of beverage and syrup applications.

Ref.	Scope	Sample	Miniaturized NIR Instr.	Data-Analytical Framework ^(a)^	General Remarks (Applicability/ Performance)
[173]	Performance evaluation of benchtop vs. portable NIR in qualitative and quantitative analysis of main sugars in syrup formulations	116 samples (53 standard and 63 reformulated syrups)	microPHAZIR	PCA, PCR, SVMR, PLSR	Good performance of the microPHAZIR in a wide range of sugar concentrations; suitable for practical use in quality control in industry
[174]	Feasibility study for a miniaturized NIR spectrometer in prediction of the main carbohydrate content in syrup; evaluation of the potential for consumer use	116 syrups consisting of different flavor types	microPHAZIR	PLSR	Reliable and accessible use of miniaturized NIR spectrometers by consumers requires further development of robust spectral-processing methods that require no/minimal supervision
[175]	Feasibility study for miniaturized NIR spectroscopy in analyzing the quality index of matcha tea	105 samples of matcha tea of different grades	NIRscan Nano	PLSR, Si-PLS, GA-PLS, CARS-PLS, RF-PLS	A model strategy based on portable NIR spectroscopy was successfully developed, with a promising potential for predicting and classifying the content of polyphenols and amino acids in matcha tea
[176]	Feasibility study for miniaturized NIR spectroscopy to predict catechins and caffeine content in green and black tea	270 tea samples (135 of black tea and 135 of green tea)	NIR-S-R2; (InnoSpectra)	SVR	Successful analysis of tea quality using a miniaturized, cost-effective NIR spectrometer
[100]	Evaluation of the analytical performance of two miniaturized NIR spectrometers (compared with a benchtop one) in the analysis of caffeine and theanine content in black tea	65 samples (milled and ground) of black tea	microPHAZIR, MicroNIR 2200	PLSR	Differences in the prediction performance of caffeine and theanine when using the two instruments were associated with their sensitivity toward the characteristic absorption bands of these two constituents
[177]	Quality control of Arabica coffee using miniaturized NIR spectrometer	125 blends of coffee	MicroNIR 1700	PCA, PLSR	The MicroNIR spectrometer was deemed successful in the prediction of adulterations with minimum quantification levels; suitability to perform real-time quality control of commercial coffee samples suggested
[178]	Analysis of sugar (sucrose) contents in everyday drinks using miniaturized NIR spectroscopy	25 sucrose solutions	NIRScan Nano	OLS, SLR, MLR, SVM, RF, MPL	Successful analysis of sucrose content, with a reasonable performance by the miniaturized NIR spectrometer
[179]	Distinguishing between beers using miniaturized NIR spectroscopy	38 beers	Systems Engineering PlaScan-SH	PCA, MLR	NIR spectroscopy was promising for beer quality evaluation, both in identifying multifarious beers, including Akita beers, using PCA; and for rapid inline quality control and inspection in beer production using the quantitative MLR analysis
[180]	Miniaturized NIR spectrometers were used for classification of Japanese saké	428 different varieties of Japanese saké	Systems Engineering PlaScan-SH	PCA	The miniaturized spectrometer was demonstrated as useful in classification of Japanese saké varieties
[181]	Development of a method for miniaturized NIR spectroscopy for prediction of the concentrations of cianidanol, ferulic acid, gallic acid, l-epicatechin, phloridzin, and rutin in congou black tea	140 samples of black tea from 7 batches	NIRQuest512 (Ocean Optics)	PLSR, CARS-PLS	The results indicated that the portable NIR, combined successfully with multivariate chemometrics, offered a nondestructive technique for the rapid screening of the phenolic compounds in congou black tea

^(a)^ Abbreviations: CARS-PLS—competitive adaptive reweighted sampling–partial least squares; GA-PLS—genetic algorithm–partial least squares; MLR—multilinear regression; OLS—ordinary least squares; PCA—principal component analysis; PCR—principal component regression; PLSR—partial-least-squares regression; RF—random forest; RF-PLS—random frog partial least squares; SLR—single linear regression; Si-PLS—synergy-interval partial least squares; SVM—support vector machine; SVMR—support vector machine regression; SVR—support vector regression; MPL—multilayer perceptron.

**Table 9:** Recent research activity oriented toward miniaturized NIR spectroscopy in the area of various miscellaneous applications.

Ref.	Scope	Sample	Miniaturized NIR Instr.	Data-Analytical Framework ^(a)^	General Remarks (Applicability/ Performance)
[182]	Feasibility study of a miniaturized NIR spectrometer in the rapid authentication of adulterated paprika powder	3 types of paprika (sweet, smoked, and spicy): 315 samples; spiked with potato starch and acacia gum (0–36% *w*/*w*) and annatto (0–18% *w*/*w*)	NIRscan Nano	PLS-DA, PLSR	Good accuracy of NIRscan Nano in detecting adulterated samples and in differentiating types of adulterations (lower only for annatto, yet still adequate for screening purposes)
[73]	Development of the onsite quantitative analysis of protein content in handcrafted insect-contaning bars using miniaturized NIR spectrometers; low-level data fusion for the simultaneous use of visible/NIR and NIR cost-effective sensors	Insect-protein-enhanced fitness 40 bars, 8 of each flavor (peanut-cranberry, hazelnut-cocoa, macadamia-salted caramel and cashew, blueberry, and “Omas Apfelstrudel”	MicroNIR 1700, Tellspec Enterprise, SCiO	PCA, PLSR, GPR	The GPR method used for the calibration hyphenated enabled the handheld instruments to quantify protein content with a good accuracy; the MicroNIR performed on par with the benchtop instrument, with the Tellspec and SCiO sensors being only moderately inferior, and as evidenced by independent test-set validation; further gains in the prediction performance for consumer-graded “pocket food analyzers” were achieved by data fusion
[183]	Prediction of egg storage time at room temperature using an ultra-cost-efficient miniaturized NIR spectrometer	30 shell-intact brown poultry eggs	SCiO	PLSR, ANN	The smartphone-connected, ultra-cost-efficient NIR spectrometer was successfully validated in egg storage time assessment; the long-term reliability was optimal when combined with traditional destructive techniques
[184]	Performance evaluation of miniaturized (in comparison with a benchtop) NIR spectrometer in classifying high-oleic-acid peanuts (HOPs) and quantitation of major fatty acids	150 different peanut varieties and strains from 10 main planting provinces	MicroNIR 1700	PCA, QDA, LDA, PLS-DA	Successful distinction of the HOPs from others, as well as for the prediction of the contents of its main fatty acids using miniaturized NIR sensors; the performance was comparable with benchtop instruments
[185]	Development of a MicroNIR-based analytical method to detect the presence of lard adulteration in palm oil; transmittance mode compared to transflectance	Pure and adulterated palm oil samples (undisclosed sample count)	MicroNIR 2200	PCA, PLSR	Successful classification and quantification analysis using the MicroNIR instrument; effective discrimination between the pure and adulterated palm oils; transmittance mode yielded a better prediction model compared to transflectance
[99]	Performance evaluation of three miniaturized NIR instruments in the quantification of piperine in black pepper	66 samples; whole and milled seeds of black pepper	MicroNIR 2200, microPHAZIR	PLSR	Reliable prediction in whole seeds only using MicroNIR 2200; miniaturized spectrometers operating in a narrow spectral region had limited performance in the quantification of piperine in black pepper; the microPHAZIR acquired only the C–H stretching bands of piperine (first overtones and binary combinations), which reduced its applicability; the MicroNIR acquired more meaningful absorption bands of piperine and offered a prediction performance comparable to the benchtop instrument
[186]	Performance evaluation of a miniaturized NIR spectrometer in the classification of food powders	8 visually indistinguishable food powders: sugar, salt, cream, flour, corn, rice, bean, and potato powders	Link Square (Stratio, Inc., San Jose, CA, USA)	KNN, RF, SVM	Successful classification of food powders using miniaturized NIR spectroscopy
[187]	Feasibility study of a miniaturized NIR spectrometer in determining the nutritional parameters of pasta/sauce blends	Commercial products: 5 pasta products, 5 sauce products; for each, 5 different pasta/sauce-type blend combinations (0–100% (*w*/*w*) sauce addition)	MicroNIR 1700	PLSR	Satisfactory prediction accuracy for quantifying energy, carbohydrates, fat, fiber, protein, and sugar in the pasta/sauce meal via miniaturized NIR spectroscopy in a realistic analytical scenario
[188]	Evaluation of two handheld NIR spectrometers for onsite and real-time analysis of nutritive parameters in raw compound feed	100 samples of intact compound feeds (feed for dairy cows, piglets, laying hens, chicken, sheep, rabbits, horses, and lambs using different presentation forms (crumbs, pellets, and meals))	microPHAZIR, MicroNIR 1700	PLSR	The handheld NIR instruments were successful in estimating the changes in the individual compound feeds’ compositions at the farm level in instantaneous manner, eliminating the largely inefficient transport of the samples from the farm to the lab; similar performances by the two popular miniaturized NIR instruments
[189]	Feasibility study and performance comparison of two distinctively different miniaturized/handheld NIR spectrometers in the quantitative analysis of crude protein (CP) content in mixed forage and feedstuff composed of sweet bran, distiller’s grains, corn silage, and corn stalk	147 total—sweet bran, corn silage, corn stalks, and three types of corn distillers grains: wet distillers grain with solubles, modified distillers grain with solubles, and dry distillers grain with solubles	Tellspec Enterprise, ASD QualitySpec Trek	PCA, PLSR	Both evaluated handheld NIR instruments accurately measured forage and feed CP; suitable in screening, quality, and process-control scenarios
[190]	Study of the feasibility of using an ultra-cost-efficient miniaturized NIR spectrometer to identify cultivars of barley, chickpea, and sorghum in the context of Ethiopia	2650 grains of barley, chickpea, and sorghum cultivars	SCiO	SVM, PLS-DA	Barley, chickpea, and sorghum cultivars were identified with perfect accuracy using miniaturized NIR spectrometers in a low-cost, rapid analysis
[191]	Estimation of rice authenticity and quality in real time using an ultra-cost-efficient miniaturized NIR spectrometer	520 rice samples from different quality grades	SCiO	PCA, KNN, SVM	Rapid and nondestructive classification of rice samples according to different quality grades, geographical origins, and imported versus locally produced rice using an ultra-cost-efficient miniaturized NIR spectrometer
[192]	Investigation of coriander seed authenticity using two miniaturized NIR spectrometers	290 coriander seed samples	Flame-NIR, SCiO	PLS-DA, OPLS-DA, RF	Inferior accuracy in the case of the miniaturized (Flame-NIR and SCiO) vs. benchtop (iS50) NIR spectrometer in quantitative analysis; however, portable sensors were suggested as viable for screening purposes
[193]	Determination of several quality parameters of canola seed using a miniaturized NIR spectrometer	181 intact whole canola seeds	MicroNIR OnSite-W	PLSR	Successful prediction of several quality parameters of canola seed (e.g., oil, protein, oleic acid, iodine); however, chlorophyll content could not be accurately predicted using the handheld instrument
[194]	Analysis of total antioxidant capacity using Folin–Ciocalteu and NIR spectroscopy; the performances of 3 miniaturized NIR instruments were evaluated and compared with a benchtop FT-NIR spectrometer	77 samples comprising buckwheat, millet, and oat	microPHAZIR, MicroNIR 2200, SCiO	PLSR, OSC	All examined instruments predicted total antioxidant capacity; however, with varying accuracy
[195]	Development and optimization of different measuring strategies for two miniaturized NIR instruments in order to find the best measuring conditions for the rapid and low-cost analysis of olive oils	66 samples of commercial oils	SCiO, NeoSpectra	LDA, PCA, PLS-DA	Without any sample pre-treatment, olive oils proved to be challenging samples, especially using the NeoSpectra; successful classification of olive oil categories and olive oil vs. sunflower oil

^(a)^ Abbreviations: ANN—artificial neural network; GPR—Gaussian process regression; KNN—k-nearest neighbors; OPLS-DA—orthogonal partial-least-squares discriminant analysis; OSC—orthogonal signal correction; PCA—principal component analysis; PLS-DA—partial-least-squares discriminant analysis; PLSR—partial-least-squares regression; RF—random forest; LDA—linear discriminant analysis; SVM—support vector machine; QDA—quadratic discriminant analysis.

**Table 10:** RMSEP values for validation of an independent test set resulting from PLSR, GPR, and ANN. The best performance levels are highlighted. Reproduced (CC-BY 4.0 license) from [31].

	NIR Spectrometer	Regression		ANN			
		PLSR	GPR	Number of Hidden Neurons			
				1	2	3	4
Dried	NIRFlex N-500	**0.27**	0.31	0.35	0.36	0.32	0.39
	MPA I	**0.27**	0.32	0.39	0.35	0.39	0.33
	microPHAZIR	0.37	0.30	**0.27**	0.30	0.31	0.48
	MicroNIR 2200	0.32	0.30	0.33	**0.29**	0.33	0.33
	MicroNIR 1700 ES	0.28	0.28	**0.25**	0.33	0.30	0.34
Native	NIRFlex N-500	0.45	**0.36**	0.73	0.55	0.41	0.43
	MPA I	0.47	**0.44**	0.54	0.68	0.56	0.59
	microPHAZIR	0.54	0.60	0.59	0.53	0.58	**0.48**
	MicroNIR 2200	0.43	0.38	**0.32**	0.35	0.44	0.44
	MicroNIR 1700 ES	0.50	**0.43**	0.46	0.70	0.67	0.72

**Table 11:** The parameters of the regression models constructed for the fused spectral data from the two cost-effective miniaturized NIR spectrometers (Tellspec Enterprise sensor and SCiO sensor) for the analysis of protein content in intact and milled bars. Analyzed protein concentration range: 19.3–23.0 % (*w*/*w*). Reproduced (CC-BY 4.0 license) from [73].

	Intact		Milled	
	PLSR	GPR	PLSR	GPR
Pretreatment	SNV, SG2 (25 SP)	SNV, SG2 (25 SP)	SG1 (11 SP)	SG1 (11 SP)
*R^2^* (Cal)	0.41	0.9	0.53	0.99
*R^2^* (CV)	0.28	0.55	0.48	0.9
RMSEC (%)	0.654	0.272	0.580	0.0002
RMSECV (%)	0.723	0.574	0.620	0.263
*R*^2^ (TSV)	0.38	0.64	0.51	0.89
**RMSEP (%)**	**0.671**	**0.517**	**0.596**	**0.295**
